# Multiscale Machine Learning Prediction of Infrared Spectra of Solvated Molecules

**DOI:** 10.1021/acs.jctc.5c01959

**Published:** 2026-02-11

**Authors:** Patrizia Mazzeo, Lorenzo Cupellini, Benedetta Mennucci

**Affiliations:** Dipartimento di Chimica e Chimica Industriale, 9310 Università di Pisa, Via G. Moruzzi 13, 56124 Pisa, Italy

## Abstract

We introduce a multiscale machine-learning molecular dynamics (MD) strategy for simulating infrared spectra of solvated molecules. Our approach integrates an efficient sampling of environmental configurations with a hierarchical model that predicts forces and dipole moments as analytical derivatives of the energy, allowing IR spectra simulations from MD trajectories. Solvent effects are incorporated through a molecular mechanics (MM) representation of the environment embedded within the ML description of the solute. Applied to representative biorelated systems, the resulting ML/MM framework reproduces experimental spectra with high fidelity and accurately captures solvent-driven vibrational shifts. This approach provides a computationally efficient and robust route for describing solvent effects in vibrational spectroscopy.

## Graphical abstract

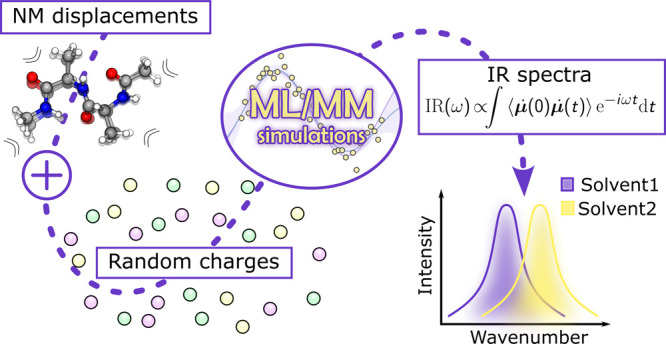

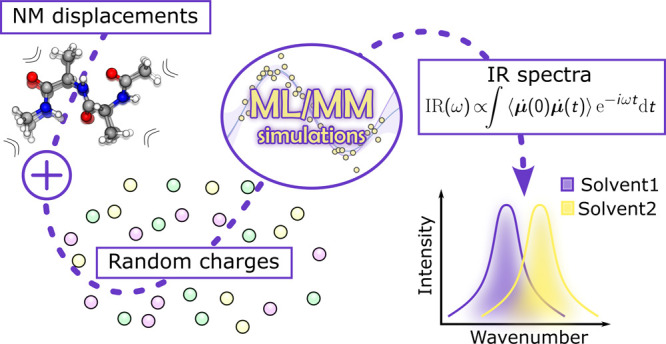

## 1 Introduction

Infrared (IR) spectroscopy provides a distinctive molecular fingerprint for identifying and characterizing chemical species, yet the growing complexity of modern experimental applications increasingly demands rigorous computational support. As advanced spectroscopic techniques probe larger and more complex systems, computational approaches have become indispensable for decoding, interpreting, and validating the measured vibrational signatures.^1–4^

The most widely used computational strategy for predicting IR spectra is based on the harmonic approximation of the potential energy surface (PES) around a local minimum obtained via geometry optimization. In this framework, vibrational frequencies and normal modes are derived from the Hessian matrix of the PES calculated with a selected quantum mechanical (QM) approach. Although this procedure can be a good starting point for IR spectra of isolated molecules, it intrinsically neglects the anharmonicities of the PES, typically accounted for by empirical scaling factors.^5,6^ Extensions to solvated systems typically rely on hybrid schemes in which a QM treatment of the solute is embedded in a continuum solvation model,^7,8^ occasionally supplemented with a small number of explicit solvent molecules to capture specific solute–solvent interactions. Such approaches, however, inherently neglect the influence of solvent dynamics, which cannot always be reliably averaged within a continuum representation.^9,10^

An alternative approach to IR spectral predictions is provided by ab initio molecular dynamics (AIMD), which explicitly incorporates nuclear motion and its impact on the infrared response.^11–14^ Also in this context, solvent effects are typically treated using a hybrid QM/classical scheme but now the environment is represented at the atomistic level through a molecular mechanics (MM) force field.^2,15–17^ Using this strategy, solvent configurations are sampled during the simulation, and the IR spectrum can be obtained from the Fourier transform of the dipole–dipole autocorrelation function. Since this procedure avoids assumptions about the PES, it implicitly captures anharmonic effects to a certain degree. The main limitation of this “dynamical” strategy is the computational cost of the MD trajectory.

In recent years, machine learning (ML) has gained popularity in the acceleration of AIMD simulations. Indeed, by replacing the QM calculation with a ML prediction, simulations can be accelerated by several orders of magnitude, while retaining a comparable accuracy to the reference level of theory adopted for the training. This paved the way to the development of ML force fields (MLFF),^18–26^ at first only for isolated molecules, and, more recently, in combination with molecular mechanics (ML/MM) to account for solvation effects through the electrostatic embedding scheme.^27–37^

Few attempts have been made to combine effective ML-based simulations with the prediction of IR spectra.^38,39^ The missing piece, in this context, is the dipole moment prediction. Early approaches focused on isolated systems and employed separate models dedicated to the dipole moment, such as a neural network (NN) in refs 40–43 and a Gaussian process regression (GPR) model in refs 44,45 . This approach evolved with the development of NNs capable of directly predicting dipole moments alongside to energies and forces.^46–49^ On this line, Gastegger et al. proposed a field-dependent NN model able to predict IR spectra in solvated systems.^50^

In previous works, we introduced an electrostatic embedding ML/MM scheme based on GPR.^28,51,52^ In this study, we extend this approach to predict solvent effect in IR spectra, evaluating dipole moments along ML/MM trajectories using analytically derived atomic partial charges.

A key challenge in this context is the data set construction: although AIMD offers optimal sampling of solvent configurations, its computational cost is substantial. For isolated molecules, variations in internal geometry are typically explored through displacements along normal modes.^20,53,54^ However, a similar procedure is not suitable for sampling solvent configurations. Furthermore, our aim is to remain solvent-agnostic, since the environment is described through the electrostatic potential and is therefore transferable—in principle—across solvents. To achieve this, we construct environment data sets by placing opportunely scaled random charges on layers built around the geometries obtained from the normal-mode displacement. These data sets can be directly employed or refined using geometries extracted from ML/MM simulations run using a preliminary model.

We apply our models and sampling strategy to compute gas-phase and solvated IR spectra of uracil, *N*-methylacetamide, and alanine dipeptide, demonstrating an accurate description of solvation effects.

## 2 Methods

A general scheme of the proposed ML/MM protocol for IR spectra in solution is shown in Figure 1. In the next subsections, we outline the methodology adopted to predict QM/MM energies, forces, and dipole moments, as well as the subsequent derivation of IR spectra from the dipole–dipole correlation. The final subsection presents the strategy developed to generate artificial solvent configurations for training the environment model.

**Figure 1:**
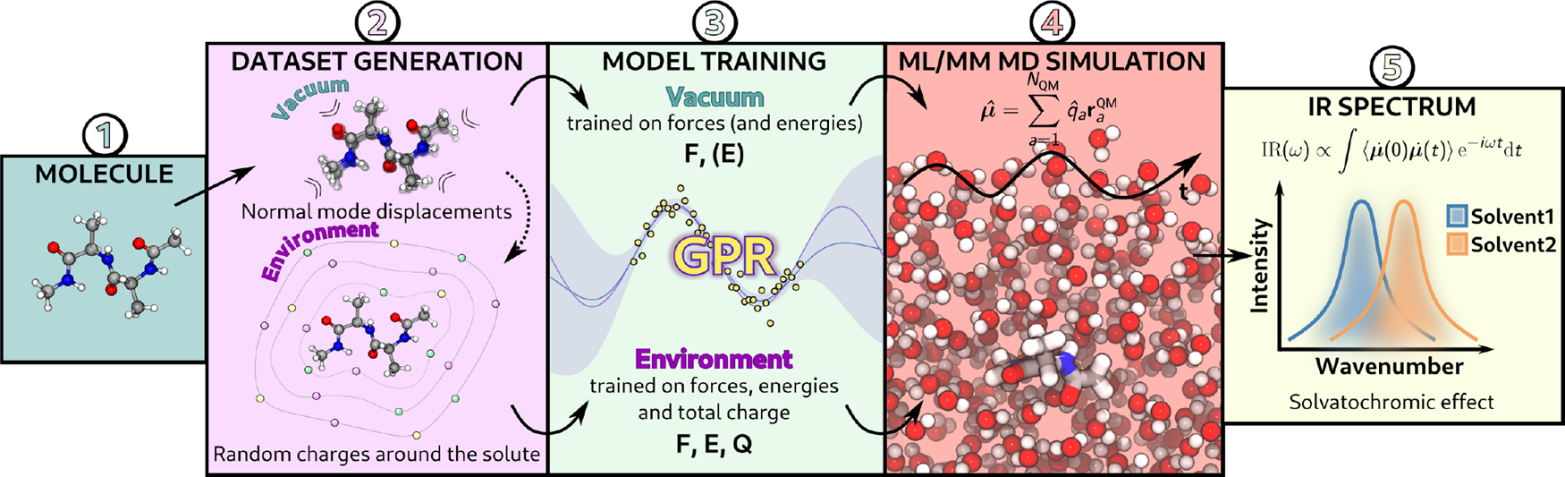
Overview of the proposed workflow. 1. Select a molecule; 2. Construct the data set for the isolated molecule via normal-mode displacements and use these isolated geometries as the basis for generating the environment data set, by placing charges on layers around the molecule; 3. Train the hierarchical model: one model for the vacuum contribution (targeting forces and, if required, energies), and another for the environment contribution (targeting forces, energies, and total charge); 4. Perform ML/MM MD simulations in different environmental conditions, predicting the dipole moment for each MD step; 5. Simulate the IR spectrum as the Fourier transform of the dipole autocorrelation function (in this case, we are using the time derivative of the dipole moment) and compare the spectra across solvents.

### 2.1 ML/MM Model for Energy, Forces, and Dipole Moment

Recently, we proposed a strategy to predict electrostatic embedding QM/MM energies and forces for ground and excited state using GPR.^28^ In this context, the QM/MM energy is written as

$$E_{\mathrm{QM}/\mathrm{MM}}=E_{\mathrm{QM}}^{\mathrm{vac}}+E_{\mathrm{QM}‐\mathrm{MM}}^{\mathrm{int}}+E_{\mathrm{QM}}^{\mathrm{pol}}=E_{\mathrm{QM}}^{\mathrm{vac}}+E_{\mathrm{QM}‐\mathrm{MM}}^{\mathrm{env}}$$ 1

where *E*_QM_^vac^ is the energy of the QM system in vacuum, *E*_QM/MM_^int^ describes the electrostatic interaction between MM charges and the QM density, and *E*_QM_^pol^ accounts for the polarization of the QM region due to the MM charges. The last two contributions are gathered into *E*_QM‑MM_^env^, representing the modifications of the QM energy due to the presence of the external environment. Our hierarchical prediction is then written as

$${\hat{E}}_{\mathrm{QM}/\mathrm{MM}}={\hat{E}}_{\mathrm{vac}}(\chi_{\mathrm{vac}},\Theta_{\mathrm{vac}})+{\hat{E}}_{\mathrm{env}}(\chi_{\mathrm{env}},\Theta_{\mathrm{env}})$$ 2

where separate models are trained for the vacuum and environment contributions, and forces are obtained as the negative gradient of this expression with respect to atomic positions. Here, **χ** denotes the descriptors, **Θ** the hyperparameters of each model, and the hat denotes model estimates.

The vacuum component only depends on the changes in the internal geometry of the QM system. Therefore, we adopted the inverse distances (ID) matrix as descriptor:

$$\chi_{\mathrm{ID},i,ab}=\frac{1}{\left||r_{i,a}^{\mathrm{QM}}-r_{i,b}^{\mathrm{QM}}|\right|}$$ 3

where *i* runs over samples, and *a*, *b* run over QM atoms. Since χ_ID,*i*,*ab*_ is symmetric for each sample *i*, only the off-diagonal elements of the matrix are retained. For this model we adopted the Matern kernel with ν = 2.5:

$$K_{\mathrm{internal}}(\chi_{\mathrm{ID},i},\chi_{\mathrm{ID},j})=\left(1+\frac{\sqrt{5}d_{ij}}{\lambda }+\frac{5d_{ij}^{2}}{3\lambda^{2}}\right)\exp\left(-\frac{\sqrt{5}d_{ij}}{\lambda }\right)$$ 4

$$d_{ij}=||\chi_{\mathrm{ID},i}-\chi_{\mathrm{ID},j}||$$

Training is performed only on forces, by solving the following linear system:

$$(\nabla_{1}^{\mathrm{QM}}\nabla_{2}^{\mathrm{QM}}K_{\mathrm{vac}}+\sigma_{\mathrm{vac}}^{2}I)\alpha_{\mathrm{vac}}=-F_{\mathrm{vac}}$$ 5

where

$$K_{\mathrm{vac}}(\chi_{i},\chi_{j})=K_{\mathrm{internal}}(\chi_{\mathrm{ID},i},\chi_{\mathrm{ID},j})$$ 6

and ∇_1_^QM^ and ∇_2_^QM^ represent the derivatives with respect to the first and the second arguments of the kernel. Thus, the Hessian is defined as

$$\nabla_{1}^{\mathrm{QM}}\nabla_{2}^{\mathrm{QM}}K_{\mathrm{vac}}(\chi_{i},\chi_{j})=\frac{\partial^{2}K_{\mathrm{vac}}(\chi (r_{i}^{\mathrm{QM}}),\chi (r_{j}^{\mathrm{QM}}))}{\partial r_{i}^{\mathrm{QM}}\partial r_{j}^{\mathrm{QM}}}$$ 7

For a new configuration χ_*_, the predicted forces and energies are

$${\hat{F}}_{\mathrm{vac}}(\chi_{*})=-\sum_{i=1}^{N_{\mathrm{samples}}}\sum_{g=1}^{3N_{\mathrm{QM}}}\alpha_{\mathrm{vac},i,g}\nabla_{1,g}^{\mathrm{QM}}\nabla_{2}^{\mathrm{QM}}K_{\mathrm{vac}}(\chi_{i},\chi_{*})$$ 8

$${\hat{E}}_{\mathrm{vac}}(\chi_{*})=\sum_{i=1}^{N_{\mathrm{samples}}}\sum_{g=1}^{3N_{\mathrm{QM}}}\alpha_{\mathrm{vac},i,g}\nabla_{1,g}^{\mathrm{QM}}K_{\mathrm{vac}}(\chi_{i},\chi_{*})+C$$ 9

where the energy is predicted as the integral of force and is therefore defined up to a constant *C*, determined on the training set $C=\sum_{i=1}^{N_{\mathrm{samples}}}\frac{E_{i}-{\hat{E}}_{i}}{N_{\mathrm{samples}}}$ .^19^

The solvent contribution in eq 1 depends on both the QM internal geometry and external MM charges. Therefore, in addition to **χ**_ID_, we introduced the potential descriptor, computed as the electrostatic potential due to external MM charges at the QM positions:

$$V_{i,a}=\sum_{m=1}^{N_{\mathrm{MM}}}\frac{q_{m}}{\left||r_{i,a}^{\mathrm{QM}}-r_{i,m}^{\mathrm{MM}}|\right|}$$ 10

where *i* runs over samples, *a* runs over QM atoms and *m* runs over MM atoms. For the *direct* effect of the solvent, we used a second-order polynomial kernel:

$$K_{\mathrm{direct}}(V_{i},V_{j})=(\theta +V_{i}^{T}\cdot V_{j}{)}^{2}-\theta^{2}$$ 11

The internal and direct kernels were multiplicatively combined to obtain the full environment kernel:

$$K_{\mathrm{env}}(\chi_{i},\chi_{j})=K_{\mathrm{direct}}(V_{i},V_{j})\cdot K_{\mathrm{internal}}(\chi_{\mathrm{ID},i},\chi_{\mathrm{ID},j})$$ 12

In the previous work,^28^ the environment model was trained on energies and forces, targeting the following quantities:

$$\begin{array}{r} F_{\mathrm{env}}=F_{\mathrm{QM}/\mathrm{MM}}-F_{\mathrm{vac}}\\ E_{\mathrm{env}}=E_{\mathrm{QM}/\mathrm{MM}}-E_{\mathrm{vac}}. \end{array}$$ 13

The corresponding linear system and predictive expressions are reported in eqs 14–16.

$$\left(\begin{array}{cc} K_{\mathrm{env}}+\sigma_{e}^{2}I & \nabla_{2}^{\mathrm{QM}}K_{\mathrm{env}}\\ \nabla_{1}^{\mathrm{QM}}K_{\mathrm{env}} & \nabla_{1}^{\mathrm{QM}}\nabla_{2}^{\mathrm{QM}}K_{\mathrm{env}}+\sigma_{f}^{2}I \end{array}\right)\left(\begin{array}{c} \alpha^{e}\\ \alpha^{f} \end{array}\right)=\left(\begin{array}{c} E_{\mathrm{env}}\\ -F_{\mathrm{env}} \end{array}\right)$$ 14

F^env(χ*)=−(∑i=1Nsamplesαie∇1QMKenv(χi,χ*)+∑i=1Nsamples∑g=13NQMαi,gf∇1,gQM∇2QMKenv(χi,χ*)) 15

E^env(χ*)=∑i=1NsamplesαieKenv(χi,χ*)+∑i=1Nsamples∑g=13NQMαi,gf∇1,gQMKenv(χi,χ*) 16

The expression we get for the energy in terms of the kernel is particularly convenient, because we can derive many physical properties as analytical derivatives of the energy. In particular, a component of the molecular dipole moment **μ** can be derived as

$$\mu_{\lambda }=-\left(\frac{\partial E}{\partial E_{\lambda }}\right)$$ 17

where λ = *x*, *y*, or *z*, and E is a uniform static external electric field. As our descriptor encodes the electrostatic potential at QM atoms, instead of an electric field, we can conveniently apply the chain rule:

$$\mu_{\lambda }=-\sum_{a=1}^{N_{\mathrm{QM}}}{\left(\frac{\partial E}{\partial V_{a}}\right)}_{V_{b\neq a}}\left(\frac{\partial V_{a}}{\partial E_{\lambda }}\right)$$

In this picture, the potential acts as a localized perturbation, and analogously to eq 17, the partial atomic charge *q*_*a*_ can be defined as

$$q_{a}={\left(\frac{\partial E}{\partial V_{a}}\right)}_{V_{b\neq a}}$$ 18

where the derivative is taken while the potentials at all other atomic sites are kept fixed, thereby isolating the local electrostatic response associated with that atom. Assuming a standard formulation for a potential derived from a uniform electric field $V_{a}=-E\cdot r_{a}$ , the dipole moment can be written as

$$\mu =\sum_{a=1}^{N_{\mathrm{QM}}}q_{a}r_{a}^{\mathrm{QM}}$$ 19

According to eq 2, the potential descriptor enters only in the environment contribution to the energy, therefore the partial charges are determined only by the environment model. From eq 18, it must also hold that ∑ _*a* = 1_^*N*_QM_^ *q*_*a*_ = *Q*, which is the total charge of the QM moiety.

Building upon eq 14, and to further impose the charge constraint ∑_*a*_ *q*_*a*_ = *Q* during training, we include the total charge in the training, associated with a very small regularization parameter (σ_*q*_ = 10^–5^). The linear system to be solved for the environment model becomes

$$(\begin{array}{ccc} K_{\mathrm{env}}+\sigma_{e}^{2}I & \nabla_{2}^{\mathrm{QM}}K_{\mathrm{env}} & \underset{b}{\sum }\partial_{2,b}K_{\mathrm{env}}\\ \nabla_{1}^{\mathrm{QM}}K_{\mathrm{env}} & \nabla_{1}^{\mathrm{QM}}\nabla_{2}^{\mathrm{QM}}K_{\mathrm{env}}+\sigma_{f}^{2}I & \nabla_{1}^{\mathrm{QM}}\underset{b}{\sum }\partial_{2,b}K_{\mathrm{env}}\\ \underset{a}{\sum }\partial_{1,a}K_{\mathrm{env}} & \underset{a}{\sum }\partial_{1,a}\nabla_{2}^{\mathrm{QM}}K_{\mathrm{env}} & \underset{a}{\sum }\partial_{1,a}\underset{b}{\sum }\partial_{2,b}K_{\mathrm{env}}+\sigma_{q}^{2}I \end{array})\times \left(\begin{array}{c} \alpha^{e}\\ \alpha^{f}\\ \alpha^{q} \end{array}\right)=\left(\begin{array}{c} E_{\mathrm{env}}\\ -F_{\mathrm{env}}\\ Q \end{array}\right)$$ 20

where $\sum_{a}\partial_{1,a}K_{\mathrm{env}}(\chi_{i},\chi_{j})=\sum_{a=1}^{N_{\mathrm{QM}}}\frac{\partial }{\partial V_{i,a}}K_{\mathrm{env}}(\chi_{i},\chi_{j})$ . For this new model, the predictions for energy, forces, and partial charges are given by

$${\hat{F}}_{\mathrm{env}}(\chi_{*})=-\left(\sum_{i=1}^{N_{\mathrm{samples}}}\alpha_{i}^{e}\nabla_{1}^{\mathrm{QM}}K_{\mathrm{env}}(\chi_{i},\chi_{*})+\sum_{i=1}^{N_{\mathrm{samples}}}\sum_{g=1}^{3N_{\mathrm{QM}}}\alpha_{i,g}^{f}\nabla_{1,g}^{\mathrm{QM}}\nabla_{2}^{\mathrm{QM}}K_{\mathrm{env}}(\chi_{i},\chi_{*})+\sum_{i=1}^{N_{\mathrm{samples}}}\alpha_{i}^{q}\sum_{a=1}^{N_{\mathrm{QM}}}\partial_{1,a}\nabla_{2}^{\mathrm{QM}}K_{\mathrm{env}}(\chi_{i},\chi_{*})\right)$$ 21

E^env(χ*)=∑i=1NsamplesαieKenv(χi,χ∗)+∑i=1Nsamples∑g=13NQMαi,gf∇1,gQMKenv(χi,χ*)+∑i=1Nsamplesαiq∑a=1NQM∂1,aKenv(χi,χ*) 22

q^a(χ*)=∑i=1Nsamplesαie∂2,aKenv(χi,χ*)+∑i=1Nsamples∑g=13NQMαi,gf∇1,gQM∂2,aKenv(χi,χ*)+∑i=1Nsamplesαiq∑b=1NQM∂1,b∂2,aKenv(χi,χ*) 23

The dipole moment is then computed from the partial charges as

μ^(χ*)=∑a=1NQMq^a(χ*)r*,aQM 24

Note that the environment model can also provide the dipole moment in vacuum, obtained by evaluating the partial charges in **V** = **0**.

We also introduced permutational symmetry, adopting the standard procedure with kernel methods.^19,26^ Letting **P**_*p*_ denote the permutation matrix for the *p*th possible permutation and *S* the number of permutations, the kernel is replaced by a symmetrized kernel, calculated as

$$K_{\mathrm{symm}}(\chi_{i},\chi_{j})=\frac{1}{S^{2}}\sum_{p,q=1}^{S}K(\chi (P_{p}r_{i}^{\mathrm{QM}}),\chi (P_{q}r_{j}^{\mathrm{QM}}))$$ 25

and analogously for all derivatives. For instance, the Hessian kernel becomes

$$\nabla_{1}^{\mathrm{QM}}\nabla_{2}^{\mathrm{QM}}K_{\mathrm{symm}}(\chi_{i},\chi_{j})=\frac{1}{S^{2}}\sum_{p,q=1}^{S}P_{p}^{T}\nabla_{1}^{\mathrm{QM}}\nabla_{2}^{\mathrm{QM}}K(\chi (P_{p}r_{i}^{\mathrm{QM}}),\chi (P_{q}r_{j}^{\mathrm{QM}}))P_{q}$$ 26

### 2.2 Infrared Spectrum Calculation

The infrared spectrum is obtained from the autocorrelation function of the dipole moment along the ML/MM simulation^11,55^ :

$$\mathrm{IR}(\omega )=\frac{2\pi \beta }{3cV}\int <\dot{\mu }(0)\dot{\mu }(t)>e^{-i\omega t}dt$$ 27

where β = 1/*k*_B_ *T*, *c* is the speed of light in vacuum, *V* is the volume, and <**μ̇**(0)**μ̇**(*t*)> is the autocorrelation function of the time-derivative of the dipole moment. Equation 27 assumes the use of a harmonic quantum correction for the line shape.^56^ Ideally, the autocorrelation function should be computed on an infinite trajectory. To avoid “border” effects due to the finite time scale of computational simulations, the autocorrelation is multiplied by a Gaussian function of σ = 1.0 ps. The spectra reported in the Results section are normalized.

### 2.3 Generation of Environment Configurations

To sample environment configurations, we constructed a grid of points around each selected solute molecule generated with the Merz–Singh–Kollmann scheme.^57–59^ Four layers of points were constructed around each configuration of the isolated molecule, setting the minimum distance from the molecule at 3 Å, interlayer distance at 2 Å and maximum cutoff of 11 Å. From the external point grid obtained, all points closer than 1.3 Å were discarded. The electrostatic potential of the molecule at these grid points was estimated from Mulliken charges^60^ (*q*_*a*_^QM^) extracted from vacuum calculations:

$$V_{i}^{\mathrm{QM}}≡V^{\mathrm{QM}}(r_{i}^{\mathrm{ext}})=\sum_{a=1}^{N_{\mathrm{QM}}}\frac{q_{a}^{\mathrm{QM}}}{\left||r_{a}^{\mathrm{QM}}-r_{i}^{\mathrm{ext}}|\right|}$$ 28

From this grid, 3000 points were stochastically selected with probabilities $p_{i}=\frac{\left|V_{i}^{\mathrm{QM}}\right|}{\sum_{j=1}^{N_{\mathrm{ext}}}|V_{j}^{\mathrm{QM}}|}$ , such that the retained points preferentially sampled regions of stronger electrostatic potential. Random numbers **ξ** ∈ [0, 1) were drawn, and the 3000 points corresponding to the smallest ratios ξ_*j*_/*p*_*j*_ were selected. External charges were then assigned to each grid point by sampling 3000 values from a normal distribution N(0,σ=0.2) . To suppress nonphysical interactions, a damping function was applied:

$$f_{\mathrm{damp}}(x)=\frac{2a}{\pi }\arctan\left(\frac{x}{a}\right)$$ 29

where the *a* parameter was empirically set to 0.1. In particular, we computed the final external charges as *q̃*_*i*_^ext^ = *f*_damp_(*q*_*i*_^ext^ *V*_*i*_^QM^)/*V*_*i*_^QM^, which gives a rescaled value for the charge only for strongly interacting sites. To enforce charge neutrality, the mean of the external charges was subtracted.

On these artificial solute–solvent configurations, we performed electrostatic embedding QM/MM calculations of energy and forces.

## 3 Computational Details

### 3.1 Data Set Generation

We considered three molecular systems: uracil (Ura), as a relatively simple test case; *N*-methylacetamide (NMA), which has the same number of atoms but introduces the challenge of permutational symmetry in methyl groups; and alanine dipeptide (Ala_2_), chosen as a larger and more flexible molecule (see Figure 2). For Ura and NMA, geometrical variations were obtained by applying random displacements along normal modes following a geometry optimization at the chosen reference level of theory. A vacuum calculation was then performed for each configuration. For Ala_2_, due to its conformational flexibility, we selected three stable aqueous conformers: α_R_, β, and P_II_ ^61^ (see Figure 2). We optimized the three conformers (α_R_: Φ = −77°, Ψ = −21°), β: Φ = −159°, Ψ = 163°, and P_II_: Φ = −65°, Ψ = 147°), and generated 400 geometries per conformer by applying random normal-mode displacements. External charges were assigned following the procedure explained in Section 2.3. For each molecule, we generated a data set consisting of 1200 samples, and we trained the models on 1000 samples. The Python scripts used for data set generation, as well as the data sets and trained models, are available at 10.5281/zenodo.18391996. Vacuum and QM/MM calculations were performed at the ωB97XD^62^/6-31G­(d)^63^ level of theory using the Gaussian suite of programs,^64^ and on these data sets we trained the **Vac** and **Env** models for each molecule.

**Figure 2:**
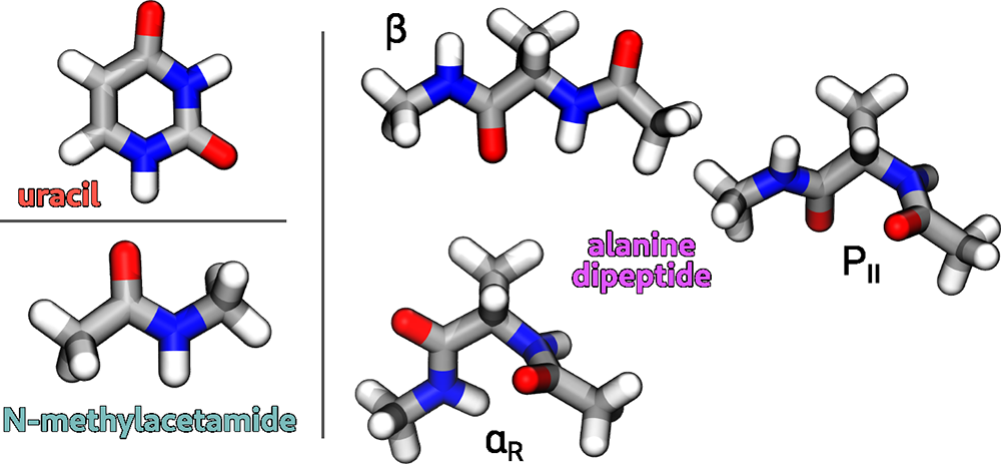
Representation of the optimized geometries for the three molecules of interest: uracil, *N*-methylacetamide, and alanine dipeptide. For the latter, we considered the α_R_, β, and P_II_ conformers.

Moreover, building on such base-model (**M** ^Base^ = **Vac** + **Env**), we adopted a Δ-learning strategy to correct the predictions targeting an higher level of theory. We chose the dispersion-corrected^65^ double-hybrid B2PLYP-D3 ^66^/*cc*-pVTZ,^67^ which was found reliable for vibrational properties.^68^ In this framework, the Δ-learning models were trained to reproduce the corrections:

$$\Delta F=F^{\mathrm{high}‐\mathrm{level}}-{\hat{F}}^{\mathrm{base}‐\mathrm{model}}$$ 30

$$\Delta E=E^{\mathrm{high}‐\mathrm{level}}-{\hat{E}}^{\mathrm{base}‐\mathrm{model}}$$ 31

where the superscript “high-level” refers to energies and forces computed at the B2PLYP-D3/*cc*-pVTZ level of theory, whereas **F̂** ^base‑model^ and *Ê* ^base‑model^ denote the predictions from the base-models. Separate Δ-learning models were trained for both the vacuum and the environment contributions. For Δ-learning models, a subset of 400 samples was extracted from the base data set and recomputed at the higher level.

Finally, for each molecule, 500 configurations were extracted from the second half of the NPT equilibration trajectory (see Section 3.5) to be used as test set and we performed the corresponding reference calculations. For the Δ-learning test set, only 250 samples were considered.

### 3.2 Training Strategy

The models developed in this work were trained using our Python package GPX.^69^ The inverse distance and electrostatic potential descriptors were computed with Moldex Python package.^70^ Both packages were implemented in JAX^71^ and are available on GitHub under the GNU LGPL agreement.

As outlined in the Methods section, our general protocol consists of training vacuum models only on forces and environment models on energies, forces, and total charge. For the latter, we enforce the same regularization parameter for energies and forces (σ = σ_*e*_ = σ_*f*_), and fix the regularization parameter for total charge at σ_*q*_ = 10^–5^. Also, the intercept parameter of the polynomial kernel was fixed at θ = 1. Thus, each model requires the tuning of two hyperparameters: the length scale λ of the Matern kernel and the regularization σ. The only exception is the vacuum model of Ala_2_, where training only on forces was insufficient to correctly discriminate between conformers in terms of energy. This was expected, since the data set contains geometries localized around the three minima and force-based training encodes only local information. Therefore, energies were included in the training, again with σ = σ_*e*_ = σ_*f*_.

The hyperparameter optimization was carried out using grid-search and 4-fold cross-validation (CV). We tested λ = [10, 20, 30] and for vacuum models σ = [10^–5^, 10^–4^, 10^–3^], while for environment models σ = [10^–4^, 10^–3^, 10^–2^]. The use of higher σ values for environment is based on prior experience, as we previously observed that too small σ values for the environment model can lead to instabilities of the system during simulation.^28^ Learning curves for all the trained models can be found in the SI (Figures S1, S10, and S13).

### 3.3 Machine Learning Scores

For the validation of the models, we report the root mean squared error (RMSE), defined as follows:

$$\mathrm{RMSE}(\hat{X})=\sqrt{\sum_{i=1}^{\overset{\sim }{N}}\frac{({\hat{X}}_{i}-X_{i}{)}^{2}}{Ñ}}X=E,F,\mu$$ 32

where *Ñ* is the number of test points (*N*) for energies, *Ñ* = 3*NN*_QM_ for forces, and *Ñ* = 3*N* for dipole moments. For dipole moments, we also report the mean absolute error (MAE), computed as

$$\mathrm{MAE}(\hat{\mu })=\frac{1}{3N}\sum_{i=1}^{3N}|{\hat{\mu }}_{i}-\mu_{i}|$$ 33

### 3.4 GPX-sire-OpenMM Interface

For performing ML/MM simulations, we extended the ML-server^72^ interface to integrate with sire,^73^ a molecular simulation framework that exploits OpenMM.^74^ This setup enables QM/MM dynamics using any external QM engine, including ML models. The interface is straightforward: sire requires only a callback function that takes as input QM coordinates, MM coordinates, and MM charges and returns energy, QM forces, and MM forces. As in our previous implementation with sander,^75^ pure MM forces and van der Waals interactions between QM and MM are computed by the molecular dynamics engine. A key advantage of the OpenMM-based approach is the GPU acceleration of the dynamics, which significantly improves performance given that the GPX software^69^ is written in JAX.^71^ Timings for all the performed simulations can be found in the SI, Table S1.

### 3.5 Molecular Dynamics Simulations

For Ura and NMA, MD inputs were prepared from optimized molecular geometries solvated in a truncated octahedron box of TIP3P^76^ water, extending 15 Å from the solute. In all simulations, TIP3P water was employed as a flexible model. We also solvated NMA with a truncated octahedron box of chloroform,^77^ extending 20 Å from the solute. We then performed a ML/MM minimization, followed by a 50 ps NPT equilibration at 300 K and 1 atm, using the Langevin thermostat (friction coefficient of 1 ps^–1^) and the Monte Carlo barostat.^78^ Production ML/MM simulations were carried out for 1 ns in the NVT ensemble using the Langevin thermostat. Electrostatics among MM charges was treated using Particle Mesh Ewald method,^79^ applying a cutoff of 12 Å, whereas all MM charges were explicitly included in the ML/MM electrostatic interaction. For simulations in vacuum, we performed a ML minimization, then a 50 ps NVT equilibration at 10 K for Ura and 20 K for NMA (resembling the conditions of experimental reference spectra), using the Langevin thermostat (friction coefficient of 0.1 ps^–1^). Production ML simulations were carried out for 1 ns in the NVT ensemble using the Langevin thermostat with the same friction coefficient adopted for the equilibration.

For Ala_2_, we considered two solvents: water and DMSO. Starting from the α_R_ and P_II_ optimized geometries employed for the training set generation, we constructed two octahedron boxes of TIP3P^76^ water (extending 15 Å from the solute), and DMSO^80^ molecules (extending 22 Å from the solute). The difference in box dimensions was chosen to yield a comparable number of MM atoms in each system. As for solvated Ura and NMA, we prepared the system through ML/MM minimization, followed by ML/MM NPT equilibration at 300 K and 1 atm for 50 ps. Subsequently, we ran 10 NVT replicas of 100 ps for each solvent and each conformation, retaining only those trajectories that preserved the corresponding conformation for IR spectrum generation.

Unless specified differently, all simulations were performed with sire (OpenMM) coupled to ML-server.

## 4 Results and Discussion

### 4.1 Uracil

After training on the artificial data set described in the Computational details section, we tested the vacuum and environment model on structures obtained from MD simulations of uracil in water (see Section 3.1).

Table 1 shows that the ML/MM model achieves robust accuracy, with prediction errors of roughly 2.3 kcal·mol^–1^ for energy and 1.6 kcal·mol^–1^·Å^–1^ for forces. An analysis of the individual contributions reveals that the dominant source of deviation stems from the environment model, which was trained on artificial charge configurations and subsequently transferred to water. Despite this, the dipole-moment error remains remarkably low (0.07 au), underscoring the reliability of the overall description.

**Table 1:** Errors on Energy, Forces, and Dipole Moment Calculated on a Test Set of Aqueous Uracil^a^

	RMSE		
property	**Vac**	**Env**	**Tot**
energy (kcal·mol^–1^)	0.02	2.30	2.29
forces (kcal·mol^–1^·Å^–1^)	0.27	1.55	1.58
dipole gas-phase (a.u.)	0.01		
dipole environment (a.u.)	0.07		

a The second, third, and fourth columns indicate the error of the vacuum model (**Vac**), environment model (**Env**), and of the total ML/MM model (**Tot** = **Vac** + **Env**). In the bottom part, we report errors on the gas-phase and solvated dipole moments predicted with the **Env** model. The reference level of theory is ωB97XD/6-31G­(d).

Figure 3 shows the correlation plot of the dipole moment, where Ura is rotated such that its molecular plane lies parallel to the *x*–*y* plane. The predictions on the μ_*x*_ and μ_*y*_ components show a strong correlation with the reference values, whereas the μ_*z*_ component is less accurately reproduced. This behavior is expected, as uracil is essentially planar. Deviations of the dipole arise only from environment-induced polarization. Because the dipole is approximated using charges restricted to the molecular plane, the out-of-plane component induced by the external charges cannot be properly captured. Nonetheless, this limitation is not expected to significantly affect the IR spectra.

**Figure 3:**
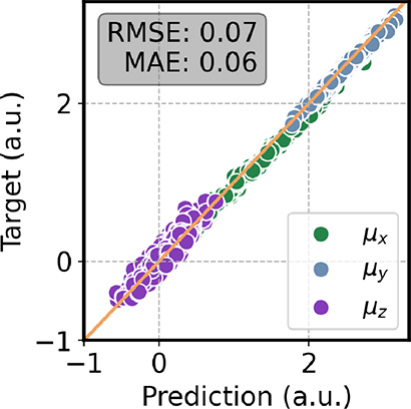
Correlation plot of the dipole moment computed on a test set of aqueous uracil. All the molecules were rotated to place the plane of uracil parallel to the *x*–*y* plane. The reference level of theory is ωB97XD/6-31G­(d).

To test the model, we performed a first comparison between the IR spectrum obtained from a QM/MM simulation and the ML/MM simulation, using the same reference QM method adopted for training. Both simulations were initiated from identical coordinates and velocities and propagated for 10 ps using sander in combination with Gaussian and ML-server, to ensure that they were performed under exactly the same conditions.

Figure 4 reports a direct comparison of the two IR spectra.

**Figure 4:**
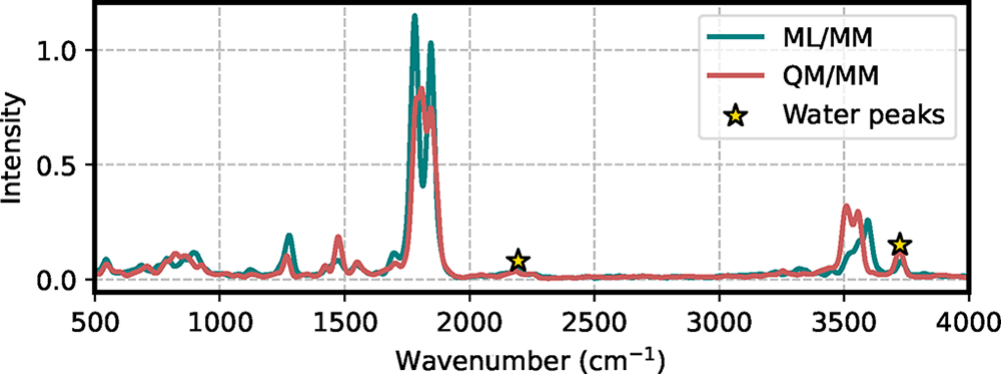
Comparison of IR spectra of aqueous uracil computed with QM/MM (red) and ML/MM (teal) molecular dynamics. The starred peaks indicate vibrations of the TIP3P water solvent.

As we discussed in our previous work,^28^ even starting from the same point in the phase space, QM/MM and ML/MM trajectories will diverge after a few hundreds of femtoseconds, since small differences in forces can lead to not negligible structural deviations. Nevertheless, we observed that the frequencies of the geometrical oscillations remain comparable, which is also evident here: the spectra are essentially superimposable in terms of vibrational frequencies. The differences observed in peak intensities can be ascribed to the relatively short simulation time scale, which may cause the energy to be unevenly distributed among the normal modes in the two trajectories. To analyze the accuracy of the dipole predictions, we recomputed the IR spectrum along the QM/MM trajectory but using ML-predicted dipole moments (see Figure S7 of the SI). This spectrum closely matches the spectrum obtained with QM dipole moments, demonstrating the accuracy of ML prediction of the dipole moment. The power spectra of uracil for the ML/MM and QM/MM simulations are presented in Figure S3 of the SI. From this plot, the peaks at ∼2200 and ∼3800 cm^–1^, which appear in both QM/MM and ML/MM spectra, are not visible. These frequencies can be attributed to the water solvent, modeled here with a flexible TIP3P model (see Figure S4 in the SI). Although the dipole of water molecules is not considered in the calculation of the IR spectrum, our ML/MM model explicitly accounts for the polarization of the ML (QM) region due to the MM part. Consequently, fluctuations in the environment induce corresponding fluctuations in the uracil dipole moment, which are then reflected in the IR spectrum. Due to the unphysical positioning of water stretching and bending modes using TIP3P as flexible, we performed a simulation of aqueous uracil using the flexible SPC/Fw water model. As shown in Figure S5 of the SI, the SPC/Fw model shifts both solvent peaks toward lower wavenumbers; specifically, the peak at 2200 cm^–1^ vanishes, and the stretching mode appears at ∼3650 cm^–1^, offering a more realistic spectral representation. The power spectrum (Figure S4 in the SI) reveals the bending mode at ∼1500 cm^–1^, where it overlaps with uracil vibrations. Notably, the uracil-related peaks remain consistent across both water models, so we retained the TIP3P simulations, as the separation between solvent and solute vibrations allowed for a more straightforward interpretation.

As described in the Computational details, we also implemented a Δ-learning scheme to refine the predictions toward a higher level of theory (here, B2PLYP-D3/*cc*-pVTZ). Table 2 presents the resulting errors in energies, forces, and dipole moments obtained with this correction strategy (see eq 30). The errors are slightly higher than those obtained with the base-models, and this can be primarily attributed to the Δ**Env** model.

**Table 2:** Errors on Energy, Forces, and Dipole Moment Calculated on the Test Set of Aqueous Uracil Computed with **M** ^Base^+Δ-models^a^

	RMSE		
property	Vac + ΔVac	Env + ΔEnv	Tot + ΔTot
energies (kcal·mol^–1^)	0.02	2.86	2.86
forces (kcal·mol^–1^·Å^–1^)	0.25	1.86	1.89
dipole gas-phase (a.u.)	0.03		
dipole environment (a.u.)	0.10		

a The second, third, and fourth columns indicate the error of vacuum models (**Vac + Δ** **Vac**), environment models (**Env + Δ** **Env**), and of the total ML/MM model (**Tot + Δ** **Tot** = **Vac** + **Env** + **Δ** **Vac** + **Δ** **Env**). In the bottom part, we report errors on the gas-phase and solvated dipole moments predicted with the **Env** + **Δ** **Env** model. The reference level of theory is B2PLYP-D3/*cc*-pVTZ.

To assess the impact of the Δ**Env** model on the IR spectra, we performed two sets of ML/MM MD simulations of 1 ns. In the first, the Δ-learning correction was applied to both the vacuum and environment models, while in the second only the vacuum model was corrected with Δ-learning, and the environment was kept at the base-model level. Note that the corresponding QM/MM simulations at B2PLYP-D3/*cc*-pVTZ level would be extremely expensive (∼0.4 ps/day), whereas Δ-learning adds a negligible overhead to the simulations (see Table S1 in the Supporting Information). The corresponding solvated spectra for both cases are shown in Figure 5, and demonstrate that the contribution of the environment Δ-learning model is negligible.

**Figure 5:**
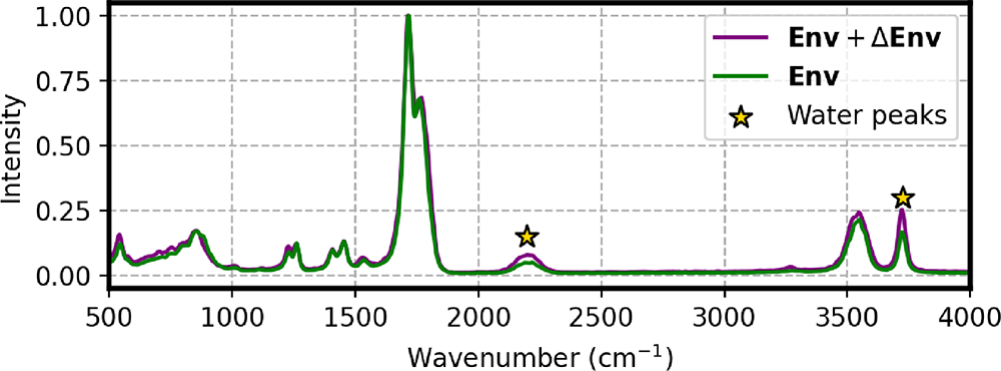
Comparison between spectra of aqueous uracil obtained from simulations in which we correct both vacuum and environment with the Δ-models (**M** ^Base^ + **ΔVac** + **ΔEnv**), and the case in which only the vacuum is corrected (**M** ^Base^ + **ΔVac**).

This finding is consistent with the small dipole-moment error already obtained using the environment base model for the B2PLYP-D3/*cc*-pVTZ reference (see Figure S2 in the SI). This observation justifies applying the correction exclusively to the vacuum model for the remaining systems. Accordingly, we define the corrected model as **M** ^Δ^ = **M** ^Base^ + **ΔVac**, where **M** ^Base^ and **M** ^Δ^ target the ωB97XD/6-31G­(d) and the B2PLYP-D3/*cc*-pVTZ levels of theory, respectively.

Since harmonic spectra represent the standard approach for computing IR, we compared the ML/MM spectrum with the harmonic spectrum evaluated at the QM/continuum level using water as solvent. The continuum model used here is the Integral Equation Formalism^81^ of the Polarizable Continumm Model (from now on PCM) as implemented in Gaussian16.^64^

The spectra reported in Figure 6 exhibit good agreement in terms of vibrational frequencies. The main discrepancies appear at ∼870, ∼2200, and ∼3800 cm^–1^. As already discussed, the latter two modes arise from the flexible TIP3P water. A geometrical analysis links the peaks in the region between 500 and 1000 cm^–1^ to uracil wagging motions. We hypothesize that the out-of-plane dipole variation along these modes may not be accurately described by our model.

**Figure 6:**
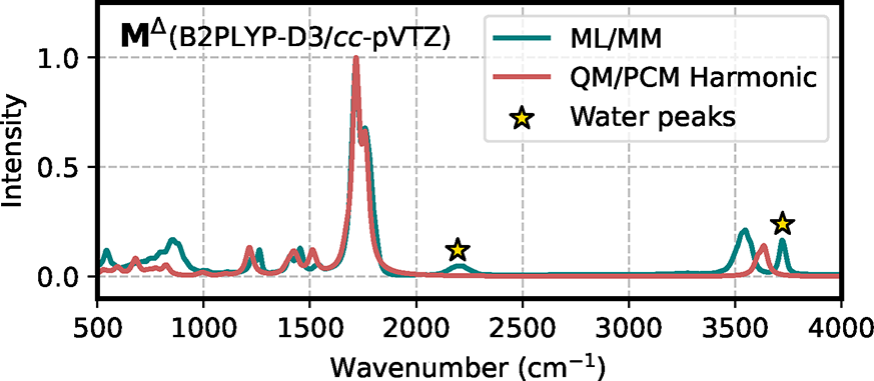
Comparison of the IR spectra of aqueous uracil obtained from ML/MM simulations and harmonic QM/PCM calculations at the B2PLYP-D3/*cc*-pVTZ level of theory.

We finally compare our results with experimental IR spectra. Figure 7 compares the gas-phase and solvated spectra calculated with the **M** ^Δ^ model to the experiments in water^83^ and in nitrogen matrix.^82^ Simulations of the isolated molecule were performed at 10 K, whereas aqueous uracil was simulated at 300 K, consistent with experimental conditions. Figure S6 in the SI compares the spectra generated from ML/MM simulations of aqueous uracil performed at 300 K using Langevin friction coefficients γ_L_ = 0.1 ps^–1^ and γ_L_ = 1 ps ^–1^. The overlapping profiles demonstrate that the higher friction coefficient γ_L_ = 1 ps ^–1^ does not introduce any artificial broadening.

**Figure 7:**
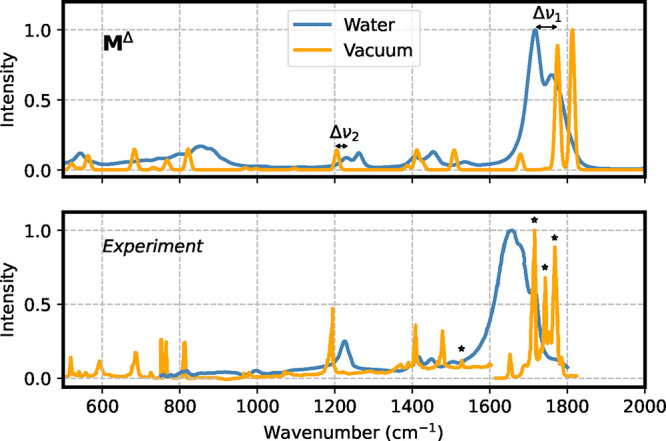
Comparison of IR spectra for gas-phase and aqueous uracil. Reference level of theory for the top panel is B2PLYP-D3/*cc*-pVTZ, whereas in the bottom panel, the experimental spectra are reported. The gas-phase spectra in different windows are taken from ref 82. and the aqueous one from ref 83. The * symbols indicate peaks that can be classified as overtones or resonance bands in the experimental vacuum spectrum.^84^

The ML-predicted spectra generally show good agreement with the experimental results. The most notable discrepancies occur in the 1600–1800 cm^–1^ region, where the experimental gas-phase spectrum displays three intense peaks instead of two, arising from the two C=O stretching modes. In this region, the uracil spectrum shows various Fermi resonances, which cause peak splitting.^84–86^ A previous computational analysis^84^ of the experimental spectrum assigned the band at ∼1761 cm^–1^ to one carbonyl group, while the other two at ∼1704 and ∼1736 cm^–1^ were interpreted as combinations of the stretching vibration of the second carbonyl with other modes. This splitting complicates the quantitative evaluation of the experimental solvatochromic shift, as it prevents a clear identification of fundamental frequencies. These effects are also present in the aqueous spectrum and may contribute to the observed broadening. For our quantitative evaluation of the shift, we considered the carbonyl peak at the lowest wavenumber (∼1704 cm^–1^ in vacuum and ∼1637 cm^–1^ in water for the experiments).

The IR spectra and the corresponding quantitative shifts obtained with **M** ^Base^ are reported in Figures S8, S9, and in Table S2 in the Supporting Information. Both models correctly reproduce the red-shift of the carbonyl stretching mode (Δν_1_ at ∼1700 cm^–1^) and the blue-shift of the C–N motion (Δν_2_ at ∼1200 cm^–1^). Using **M** ^Δ^, we obtained a red shift of 58 cm^–1^ for Δν_1_, compared to 67 cm^–1^ observed experimentally, and a blue shift of 25 cm^–1^ for Δν_2_, in good agreement with the experimental value of 30 cm^–1^. Furthermore, this higher-level model appears to provide a closer match to experimental peak shapes.

### 4.2 *N*-Methylacetamide

For *N*-methylacetamide (NMA), we trained both the base-models and the Δ**Vac** correction, similarly to the case of uracil. The additional difficulty with this molecule stems from the presence of chemically equivalent atoms in the methyl groups, which were treated using a symmetrized kernel as explained in Section 2.

Table 3 shows the errors for the vacuum and environment models, as well as for their sum. As in the previous case, the environment model exhibits a larger error, although it is still able to reproduce the gas-phase and QM/MM dipole moment with good accuracy. The last column of Table 3 shows the error of the base+Δ*v*acuum model with respect to the B2PLYP-D3/*cc*-pVTZ reference calculations in vacuum.

**Table 3:** Errors on Energy, Forces, and Dipole Moment Calculated on a Test Set of Aqueous NMA^a^

	RMSE			
property	**Vac**	**Env**	**M** ^Base^	**Vac** + Δ**Vac**
energies (kcal · mol^–1^)	0.16	1.42	1.45	0.14
forces (kcal · mol^–1^ Å^–1^)	0.61	2.17	2.18	0.62
dipole gas-phase (a.u.)		0.02		
dipole environment (a.u.)		0.05		

a The second, third, and fourth columns indicate the error of the vacuum model (**Vac**), environment model (**Env**), and of the total ML/MM model (**M** ^Base^ = **Vac** + **Env**). In the bottom part, we report errors on the gas-phase and solvated dipole moments predicted with the **Env** model. These errors are computed with respect to the reference ωB97XD/6-31G­(d) level. The last column represents the error of the base+Δ vacuum model with respect to the B2PLYP-D3/*cc*-pVTZ reference in vacuum.

In Figure 8 we report the correlation of the dipole moments predicted by the **Env** model with the B2PLYP-D3/*cc*-pVTZ calculations. Also in this case the base-model predicts quite well the dipole moment at the higher level.

**Figure 8:**
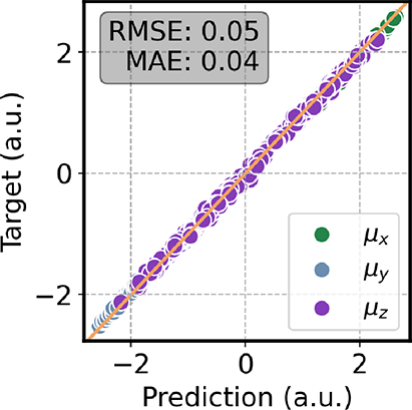
Correlation plot of the dipole moment computed on a test set of aqueous *N*-methylacetamide. The prediction is made using the **Env** model and compared with reference calculations at the B2PLYP-D3/*cc*-pVTZ level.

As a further analysis, we compare the ML/MM spectrum of aqueous NMA with the QM/PCM harmonic approach (Figure 9). To understand the effect of hydrogen bonding, we consider both pure PCM calculations and calculations including three explicit water molecules in the QM region (while the rest of the solvent is described as PCM). We included two H_2_O molecules hydrogen-bonded with the carbonyl group and one interacting with the N–H group. This is a challenging comparison: excluding explicit water neglects hydrogen bonding effects, while including them couples the water vibrational modes with those of NMA, generating peaks that include the effect of the dipole of explicit water molecules. Here we focus on the 1000–1900 cm^–1^ region. Overall, the QM/PCM harmonic spectrum is consistent with the ML/MM one, except in the carbonyl region, where the absence of hydrogen bonds causes a marked separation between the Amide I (∼1700 cm^–1^) and Amide II (∼1600 cm^–1^) bands. On the other hand, a quantum-mechanical description of the hydrogen bonds immediately close to the NMA molecule reduces the separation between the two peaks, highlighting the significant role of hydrogen bonding in the accurate description of this spectrum. The ML/MM approach yields results intermediate between the PCM-only case and the microsolvation case, suggesting that our scheme partially captures hydrogen-bonding effects but remains limited by the absence of mutual polarization between NMA and water molecules.

**Figure 9:**
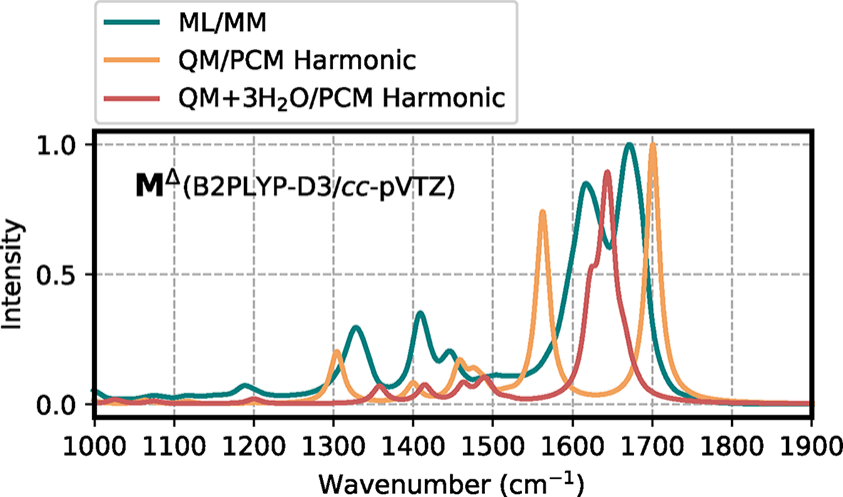
Comparison of the IR spectrum obtained from ML/MM simulations of aqueous NMA, and the harmonic spectrum in PCM water and the one with three hydrogen-bonded water molecules in the QM part. The harmonic spectra are computed at the B2PLYP-D3/*cc*-pVTZ level.

Figure 10 compares the IR spectra of NMA in gas-phase, water, and chloroform from **M** ^Δ^ model with the experiments. Simulations were performed at 20 K for vacuum and 300 K for ML/MM, also in this case matching experimental conditions.

**Figure 10:**
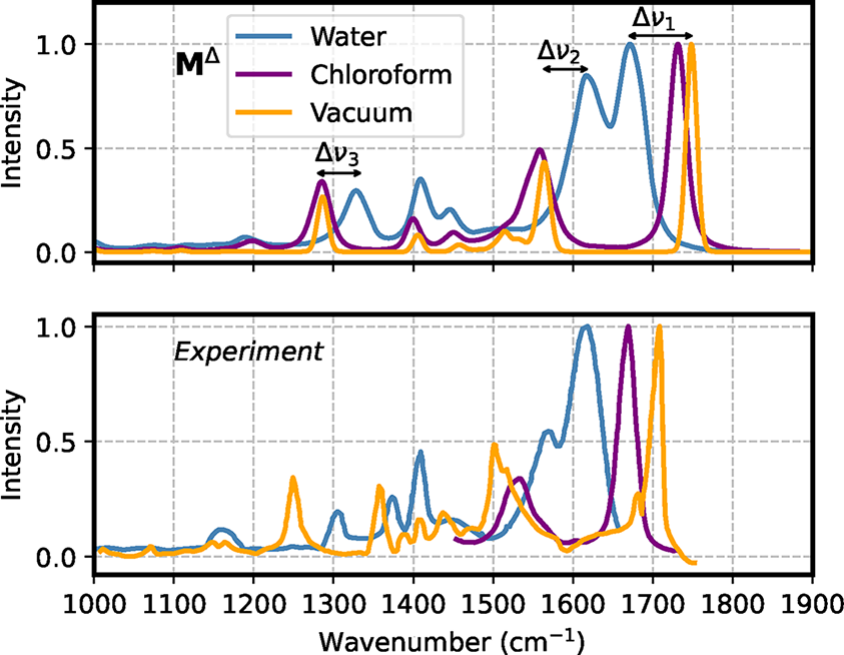
Comparison of IR spectra for NMA in gas-phase, chloroform, and water. Reference level of theory for the top panel is B2PLYP-D3/*cc*-pVTZ, whereas in the bottom panel, the experimental spectrum is reported. The gas-phase spectrum is taken from ref 87. while the aqueous and chloroform spectra are from refs 88 and 89, respectively.

The predicted spectra in water well reproduce the main solvent effects observed in the experiments: namely, a red shift of the Amide I peak and a blue shift of the Amide II and Amide III peaks (∼1600 and ∼1300 cm^–1^, respectively). A more quantitative comparison (Table 4) reveals that these shifts are slightly underestimated by ML/MM simulations. From the comparisons with PCM calculations detailed above, which highlighted the strong influence of the hydrogen-bond description on IR spectra, we can conclude that a purely electrostatic embedding does not fully capture the effect of H-bonds on these frequencies. Nonetheless, the agreement is almost quantitative. Similarly, the predicted spectrum in chloroform resembles the experimental one, although it is closer to the gas-phase spectrum than observed experimentally. The trend of the Amide I peak is correctly reproduced, and, as in water, its shift is slightly underestimated. In contrast, the Amide II and Amide III bands are almost superimposed to their vacuum counterparts. The discrepancy with the experimentally observed shift of the Amide II peak can be ascribed to the lack of solvent polarization, which plays an important role in describing the effects of low-polar solvents. Notably, the Amide II shift is difficult to determine quantitatively due to the peculiar shape of the experimental vacuum peak. IR spectra and shifts obtained with **M** ^Base^ are reported in Figures S11, S12, and in Table S3 in the Supporting Information.

**Table 4:** Solvatochromic Shift (cm^–1^) of the IR Spectra of NMA in Water and Chloroform

	water (cm^–1^)			chloroform (cm^–1^)		
	Δν_1_	Δν_2_	Δν_3_	Δν_1_	Δν_2_	Δν_3_
	(Amide I)	(Amide II)	(Amide III)			
Exp.	–90	68	57	–39	15	
**M** ^Δ^	–77	53	41	–17	–6	–1

### 4.3 Alanine Dipeptide

Also for Ala_2_, base models and Δ**Vac** model have been trained. Here, **Vac** and Δ**Vac** models are trained on energies and forces, and **Env** model is trained on energies, forces and total charge. We employed the symmetrized kernels to account for the permutational invariance of hydrogen atoms in the three methyl groups. Table 5 shows the errors of **Vac** and **Env** models, and their sum (**M** ^Base^). The test set in this case contains frames extracted from the equilibration of both α_R_ and P_II_ trajectories and for both water and DMSO solvents. In the last column of the table, the error of the **Vac**+Δ**Vac** model is also reported, with respect to the B2PLYP-D3/*cc*-pVTZ reference calculations in vacuum. Figure 11 reports the correlation plot for the dipole moment of solvated Ala_2_ using as target the B2PLYP-D3/*cc*-pVTZ calculations.

**Table 5:** Errors on Energy, Forces, and Dipole Moment Calculated on a Combined Test Set of Ala_2_ in Water and DMSO^a^

	RMSE			
property	**Vac**	**Env**	**M** ^Base^	**Vac** + Δ**Vac**
energies (kcal·mol^–1^)	0.58	1.75	1.84	0.56
forces (kcal·mol^–1^·Å^–1^)	1.33	1.93	2.33	1.32
dipole gas-phase (a.u.)		0.06		
dipole environment (a.u.)		0.11		

a The second, third, and fourth columns indicate the error of the vacuum model (**Vac**), environment model (**Env**), and of the total ML/MM model (**M** ^Base^ = **Vac** + **Env**). In the bottom part, we report errors on the gas-phase and solvated dipole moments predicted with the **Env** model. These errors are computed with respect to the reference ωB97XD/6-31G­(d) level. The last column represents the error of the base+Δ vacuum model with respect to the B2PLYP-D3/*cc*-pVTZ reference in vacuum.

**Figure 11:**
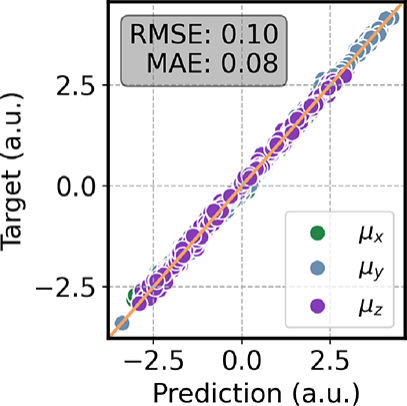
Correlation plot of the dipole moment computed on a test set of solvated Ala_2_. The prediction is made using the **Env** model and compared with reference calculations at the B2PLYP-D3/*cc*-pVTZ level.

Ala_2_ errors are slightly higher than those observed for the other two molecules, but this can be primarily attributed to the vacuum model rather than the environment model. This is not a surprise, given the conformational flexibility of Ala_2_ and the bias of the vacuum data set toward optimized geometries and their local regions. A possible way to address this issue is active learning,^90,91^ by performing an “explorative” simulation with the current models and extracting new geometries to enrich the data set. As the aim of our work is to evaluate the potential of our data set generation protocol and model independently of the solvent, we evaluate our model on ML/MM dynamics without refitting. To this end, we compare IR spectra simulated in two different solvents, DMSO and water.

The literature reports that Ala_2_ can adopt three preferential conformations in water, α_R_, β and P_II_, with their interconversion being barrierless (β to P_II_) or with a rather low barrier (β/P_II_ to α_R_).^61,92^ That is the reason why, consistently with a previous work on simulation of Ala_2_ IR spectrum using MD,^61^ we performed two sets of simulations (10 replicas each), starting from α_R_ and P_II_ conformations. The IR spectrum of α_R_ was obtained by averaging trajectories that started and remained in this conformation, while the spectrum of P_II_/β was derived from trajectories that started from P_II_ and remained in either the P_II_ or the β conformations. Notably, all replicas in DMSO remained in their respective target conformation. Figures S14 and S15 in the SI show the Ala_2_ spectra in water and DMSO for the 1000–1900 cm^–1^ region, computed from the α_R_ and P_II_/β trajectories. Also, in Figure S16 of the SI we report the 2d-histogram for the Φ and Ψ angles explored during those simulations. For comparison with experiments, the two spectra were combined with 1:5 weighting (α_R_: P_II_/β), as proposed in ref 61.

In Figure 12 we compare the IR spectra (Amide I region) obtained with **M** ^Δ^ for Ala_2_ in water and DMSO with their experimental counterparts. The model is correctly capturing the solvatochromic shift, namely – 38 cm^–1^ with respect to the measured – 29 cm^–1^. The spectra and solvatochromic shift obtained with the **M** ^Base^ model are provided in Figure S17 and in Table S4 in the Supporting Information, and show similar trends.

**Figure 12:**
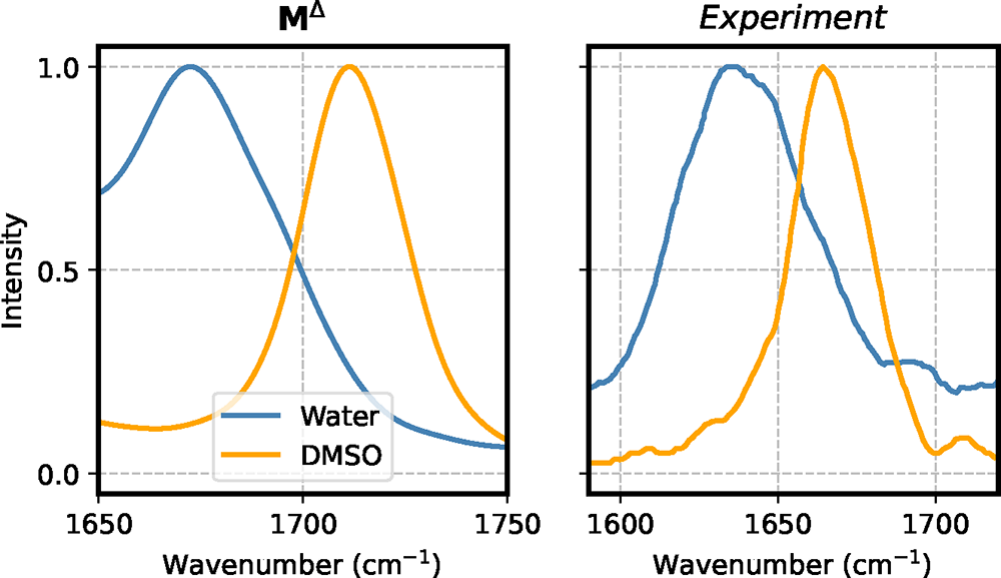
Comparison of IR spectra in the Amide I region for Ala_2_ in water and DMSO. Reference level of theory for the left panel is B2PLYP-D3/cc-pVTZ, whereas in the right panel the experimental spectrum is reported. The experimental spectra are taken from ref 93.

These results suggest that our ML/MM model can capture not only the effects of solvent polarity, but more in general the specific interactions that differentiate two polar solvents like water and DMSO. We recall that for both solvents we employed the same model, trained on artificial charge configurations. Our results thus indicate a good transferability of the ML/MM model across environments.

## 5 Conclusions

We have presented a general protocol for computing IR spectra of solvated molecules using ML/MM simulations within the electrostatic embedding framework. For any new molecule, the protocol begins with the construction of a vacuum data set, generated by applying random displacements along normal modes of optimized geometries at one or more minima. Optimally defined random charges are then placed around each isolated geometry, producing a solvent-agnostic data set suitable for environment training. Strikingly, this approach allows us to avoid solvent-specific simulations for extracting environment configurations. This strategy is completely general and would be particularly advantageous to generate large data sets, as in the case of training neural network models.^33,46,50^

In this work, both vacuum and environment model were trained using GPR, and subsequently employed for ML/MM simulations, where the environment model provided partial charges for dipole moment predictions at each MD step. The IR spectrum was then obtained as the Fourier transform of the time-derivative of the dipole–dipole correlation function.

Across all studied systems, our protocol successfully reproduced experimental solvatochromic shifts. Notably, the environment model could distinguish not only between two significantly different environments, such as gas-phase, chloroform, and water (as demonstrated for Ura and NMA), but also between two polar solvents (water and DMSO for Ala_2_) despite being trained without solvent-specific data. This highlights that the electrostatic potential serves as an effective, though approximate, descriptor for encoding the electrostatics of the environment. Furthermore, applying Δ-learning to correct only the vacuum model results in a negligible increase of the computational cost while yielding a clear improvement in the simulated IR spectra. The methodology presented here is particularly useful for studying the effect of different solvents on the same molecule, as kernel methods allow efficient model training with limited data (1000 samples per model in our case).

Future developments could involve more refined environment representations. For instance, incorporating the electric field in addition to the electrostatic potential would provide directional information about the environment. A current limitation is that kernel methods are molecule-specific. A significant future goal is the development of more general frameworks capable of incorporating environmental effects across multiple molecular systems.

## Supplementary Material



## Data Availability

All data and scripts useful for this work are available in a Zenodo repository at 10.5281/zenodo.18391996. In particular, we have provided the data sets with both levels of theory, the Python scripts for generating new geometries (including normal-mode displacements and artificial environment configurations), the trained models for the three studied molecules, and the dipole moment data used for the IR spectra computations. Software: ML-server: https://github.com/Molecolab-Pisa/ML-server; GPX: https://github.com/Molecolab-Pisa/GPX (permut_symm branch); Moldex: https://github.com/Molecolab-Pisa/moldex.

## Notes

Learning curves for all the trained models (uracil, NMA, and alanine dipeptide); correlation plot of the dipole moment computed with the base-models and compared with the B2PLYP-D3/*cc*-pVTZ level of theory (uracil); power spectra computed on QM/MM and ML/MM simulations (uracil); IR spectra and power spectra of a TIP3P and SPC/Fw water molecules during ML/MM simulations (uracil); IR spectra obtained with ML/MM simulations using two different Langevin friction coefficients (uracil); comparison between the IR spectra obtained from QM/MM and ML/MM dipole moments on the QM/MM trajectory (uracil); comparison of the IR spectra obtained with the base-models and Δ-learning corrected models with the corresponding harmonic spectra in PCM (uracil and NMA); IR spectra obtained with the base-models and Δ-learning corrected models averaged on α_R_ and P_II_ trajectories in water and DMSO (alanine dipeptide); 2d-histogram of the values of Ramachandran angles during the ML/MM simulations performed in water and DMSO­(alanine dipeptide); comparison of the IR spectra obtained with the base-models and Δ-learning corrected models with the corresponding experimental spectra (uracil, NMA and alanine dipeptide); additional tables: timings for the performed ML/MM simulations; and quantitative solvatochromic shifts for base-models and Δ-learning corrected models compared with experimental ones for the three molecules (PDF)
