# Supplementary material for: Multiscale Machine Learning Prediction of Infrared Spectra of Solvated Molecules
Source: J Chem Theory Comput. 2026 Feb 11;22(4):1883–95. doi: 10.1021/acs.jctc.5c01959 (PMC12937103; doi:10.1021/acs.jctc.5c01959)
Supplement: Supplementary file 1 [file ct5c01959_si_001.pdf]

Supporting Information:
Multiscale machine learning prediction of infrared spectra of solvated molecules

Patrizia Mazzeo[a], Lorenzo Cupellini[a,*], and Benedetta Mennucci[a]

[a]Dipartimento di Chimica e Chimica Industriale, Università di Pisa, Via G. Moruzzi 13, 56124 Pisa, Italy
[*]Corresponding author. E-mail: lorenzo.cupellini@unipi.it

S1 Additional figures

S1.1 Uracil

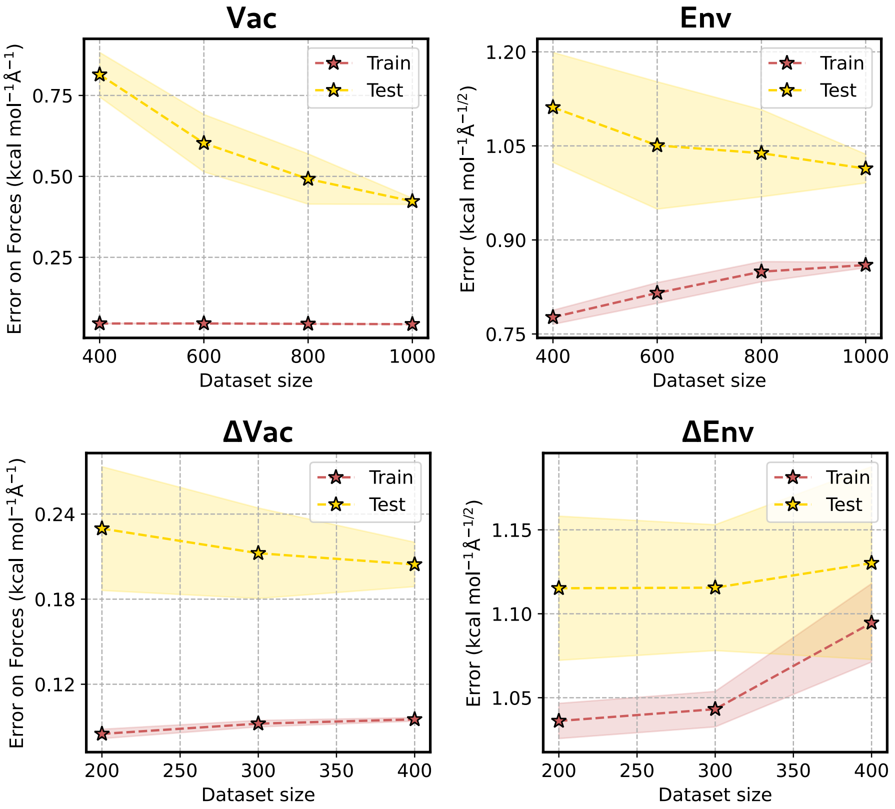

Figure S1: Learning curves for all the models trained on uracil. For the two vacuum models the RMSE is evaluated only on forces, while for the two environment models, the error is the geometric mean of RMSE on forces and energies. Results are obtained from four-fold CV: stars indicate the mean error across folds and the shaded regions denote the standard deviation.

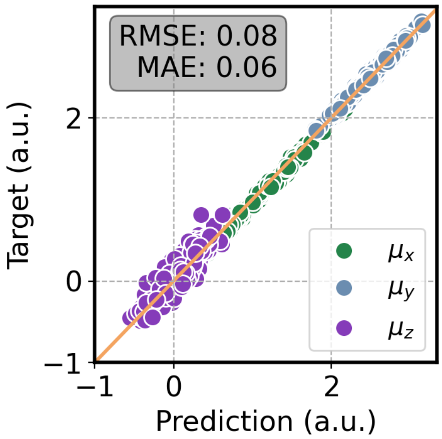

Figure S2: Correlation plot of the dipole moment computed on a test set of aqueous uracil. The prediction is made using the **Env** model and compared with reference calculations at the B2PLYP-D3/*cc*-pVTZ level. All the molecules were rotated to place the plane of uracil parallel to the x-y plane.

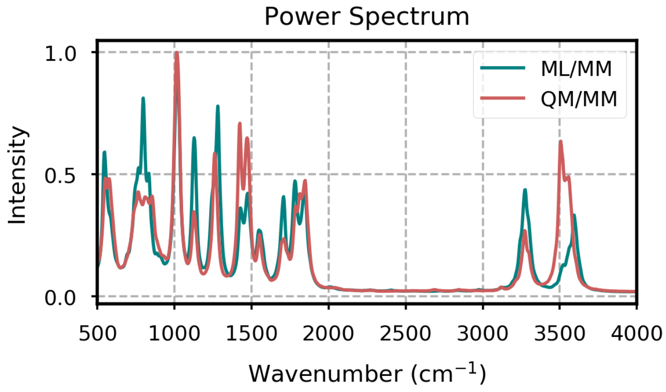

Figure S3: Comparison of power spectra for uracil obtained from ML/MM and QM/MM simulations (in water), both initialized from the same starting conditions.

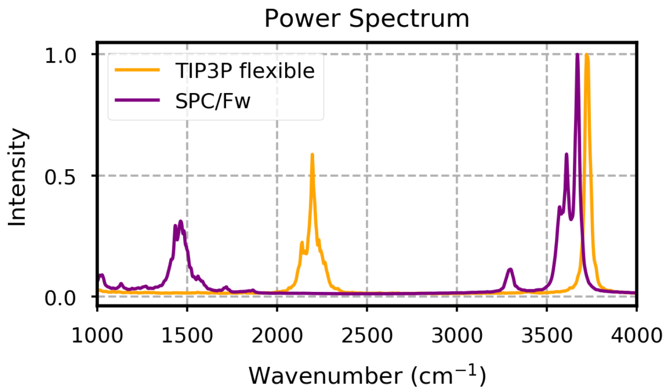

Figure S4: Power spectra computed on a flexible TIP3P and a SPC/Fw water molecule from the corresponding ML/MM simulations of aqueous uracil.

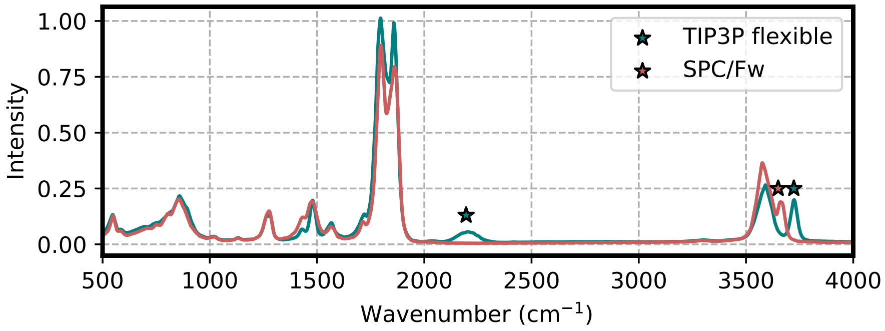

Figure S5: IR spectra of aqueous uracil using flexible TIP3P and SPC/Fw water models.

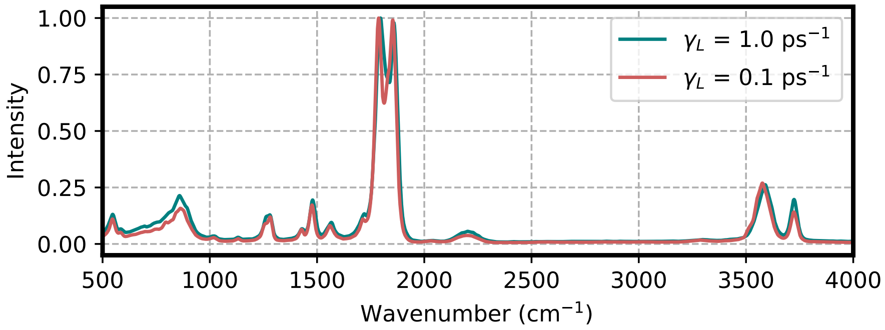

Figure S6: IR spectra of aqueous uracil using two different Langevin friction coefficients ($\gamma_L = 1$ ps^{-1} $\gamma_L = 0.1$ ps^{-1}) in the ML/MM simulations.

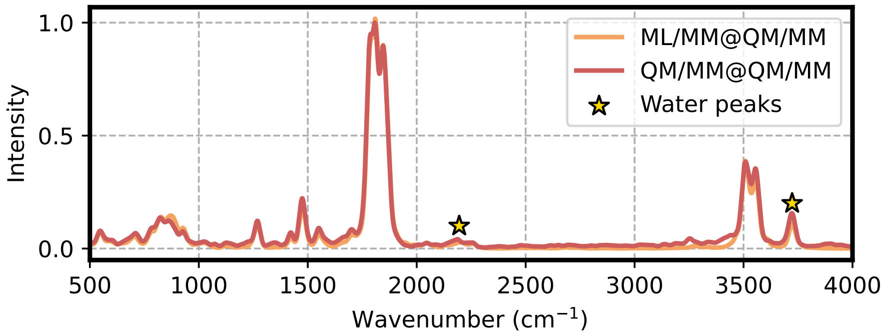

Figure S7: Comparison between the IR spectrum of aqueous uracil obtained with ML-predicted dipole moment (orange) and QM dipole moment (red) using the same QM/MM simulation frames. The stars indicate peaks that are attributed to the TIP3P water molecules.

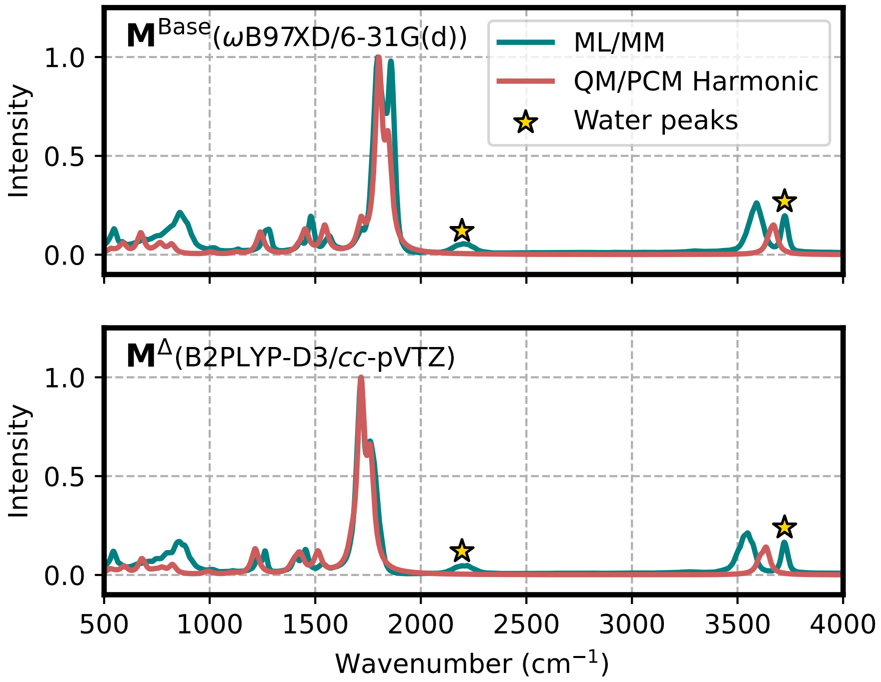

Figure S8: Comparison of the IR spectra obtained from ML/MM simulations of aqueous uracil and harmonic spectra in PCM water. The spectrum obtained with $\mathbf{M}^{\text{Base}}$ is compared to the harmonic spectrum at the ωB97XD/6-31G(d) level, while the spectrum obtained with $\mathbf{M}^{\Delta}$ is compared to the harmonic spectrum at the B2PLYP-D3/*cc*-pVTZ level.

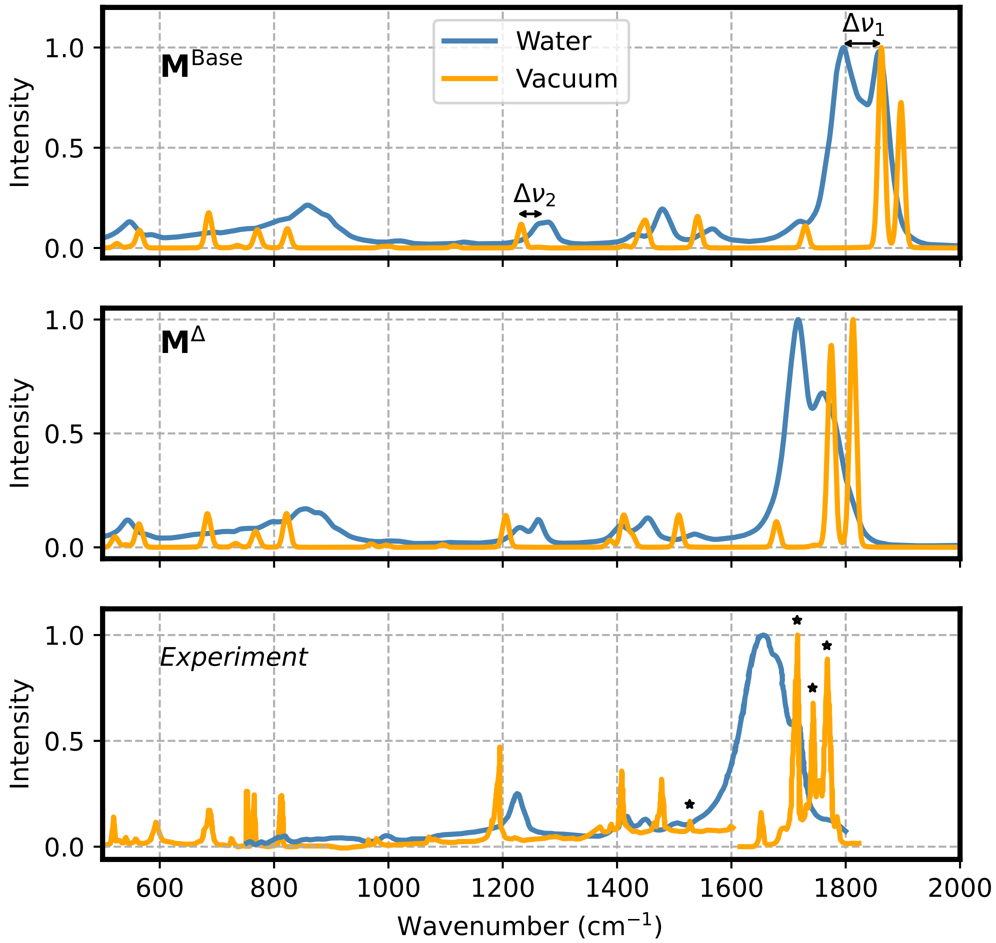

Figure S9: Comparison of IR spectra for gas-phase and aqueous uracil. Reference level of theory for the top and middle panels are ωB97XD/6-31G(d) and B2PLYP-D3/*cc*-pVTZ, respectively, whereas in the bottom panel the experimental spectra are reported. The gas-phase spectra in different windows are taken from Ref. 1 and the aqueous one from Ref. 2. The * symbols indicate peaks that can be classified as overtones or resonance bands in the experimental vacuum spectrum.

S1.2 N-methylacetamide

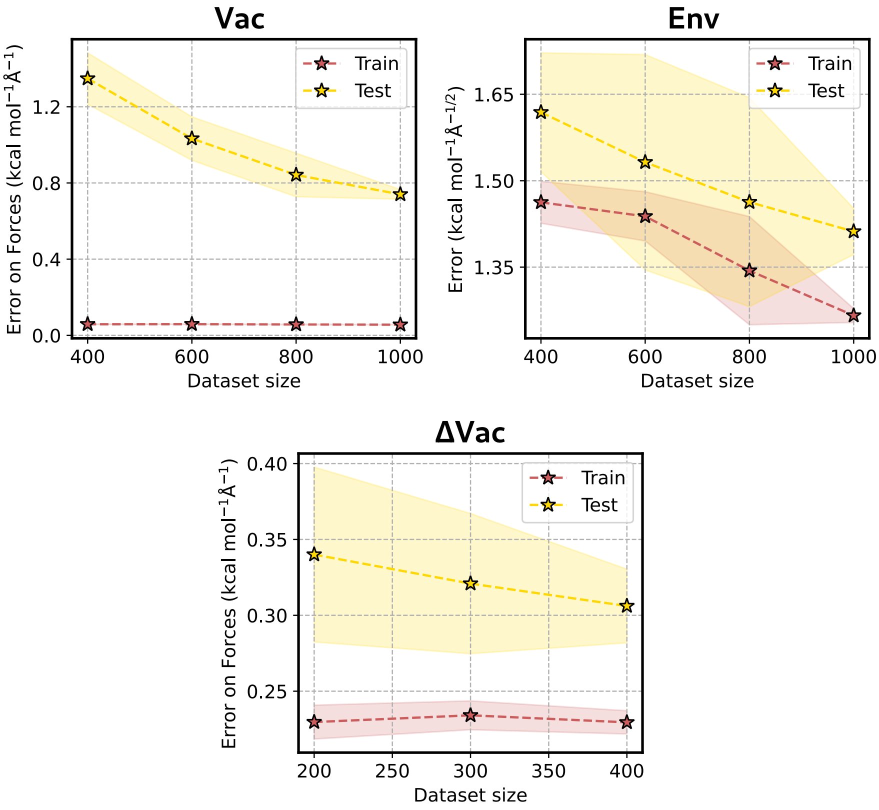

Figure S10: Learning curves for all the models trained on N-methylacetamide. For the two vacuum models the RMSE is evaluated only on forces, while for the environment model, the error is the geometric mean of RMSE on forces and energies. Results are obtained from four-fold CV: stars indicate the mean error across folds and the shaded regions denote the standard deviation.

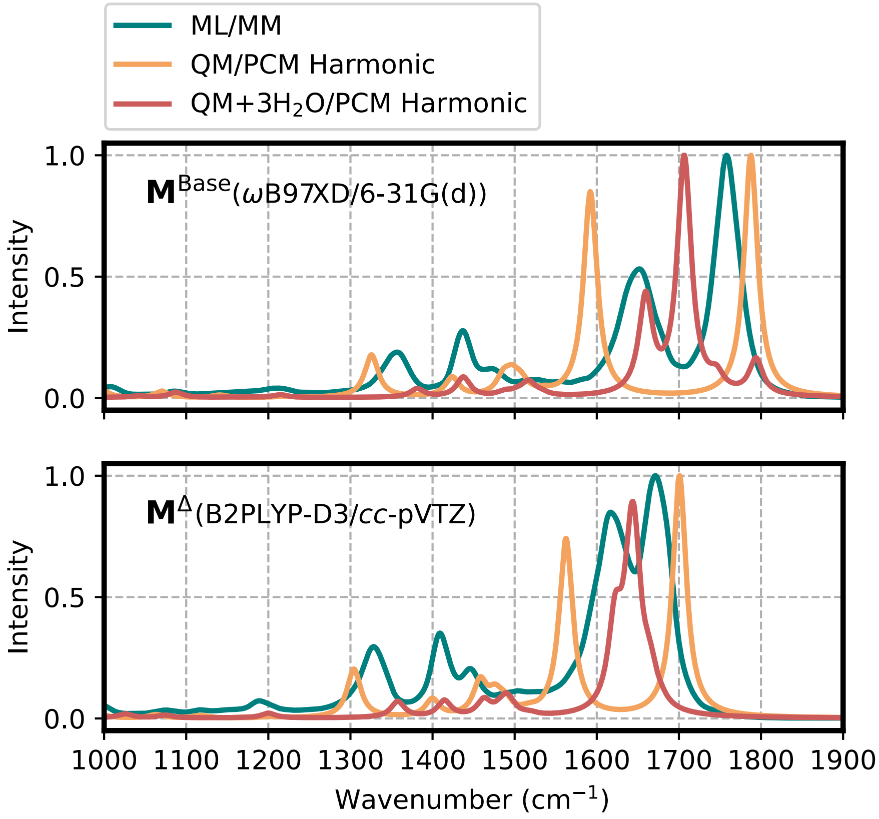

Figure S11: Comparison of the IR spectra obtained from ML/MM simulations of aqueous NMA, and the harmonic spectra in PCM water and with three hydrogen-bonded water molecules in the QM part. The spectrum generated using $\mathbf{M}^{\mathrm{Base}}$ is compared to the harmonic spectra at the ωB97XD/6-31G(d) level, whereas the spectrum obtained with $\mathbf{M}^{\Delta}$ is compared to the harmonic spectra at the B2PLYP-D3/*cc*-pVTZ level.

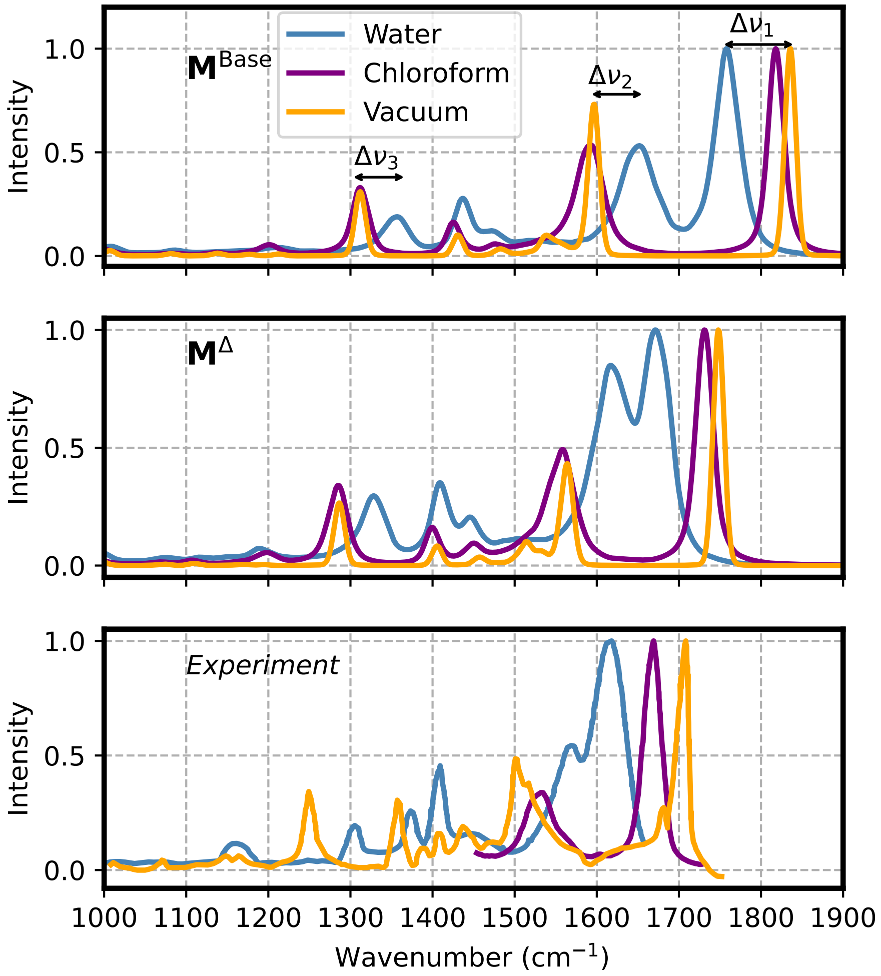

Figure S12: Comparison of IR spectra for NMA in gas-phase, chloroform and water. Reference level of theory for the top and middle panels are ωB97XD/6-31G(d) and B2PLYP-D3/*cc*-pVTZ, respectively, whereas in the bottom panel the experimental spectrum is reported. The gas-phase spectrum is taken from Ref. 3 while the aqueous and chloroform spectra are from Ref. 4 and Ref. 5, respectively.

S1.3 Alanine dipeptide

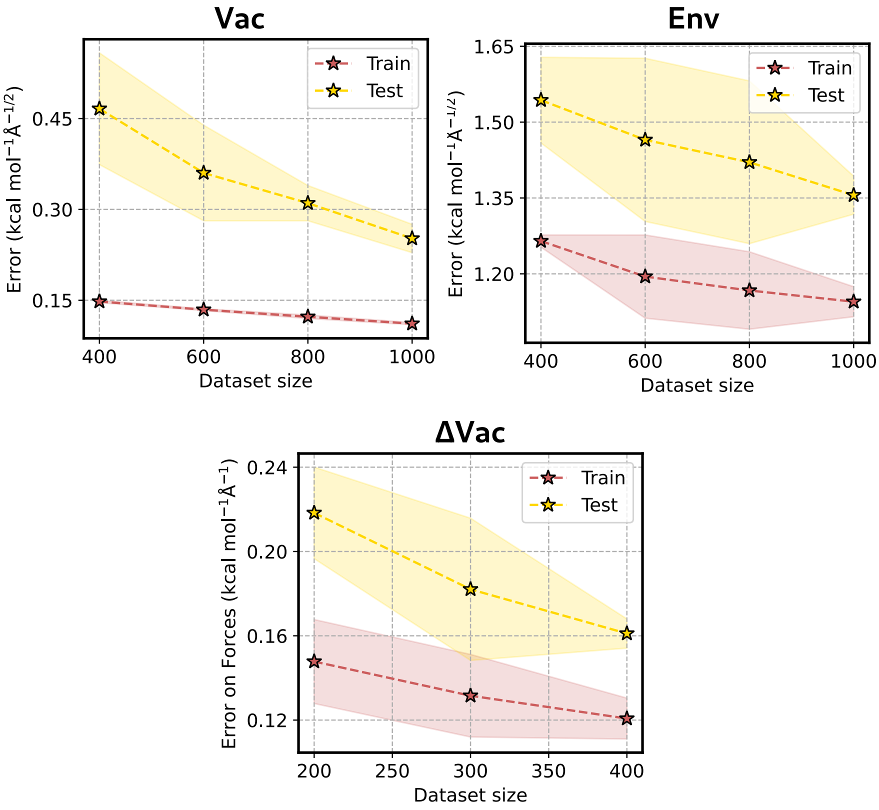

Figure S13: Learning curves for all the models trained on Ala_2. For all the models, the error is evaluated as the geometric mean of RMSE on forces and energies. Results are obtained from four-fold CV: stars indicate the mean error across folds and the shaded regions denote the standard deviation.

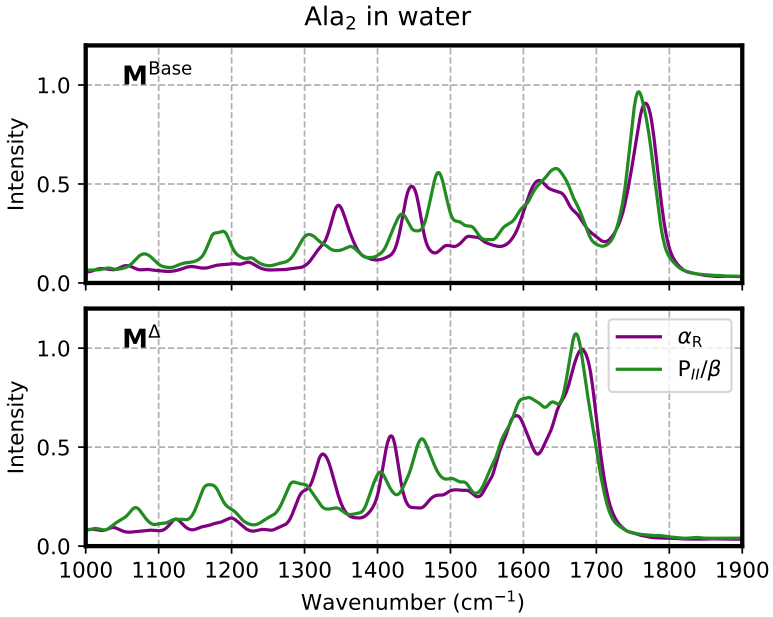

Figure S14: IR spectra in the 1000-1900 cm^{-1} region of aqueous Ala$_2$ obtained from trajectories that remained in the α_R and P_{II}/β conformations. Reference level of theory for the top and bottom panels are ωB97XD/6-31G(d) and B2PLYP-D3/*cc*-pVTZ, respectively.

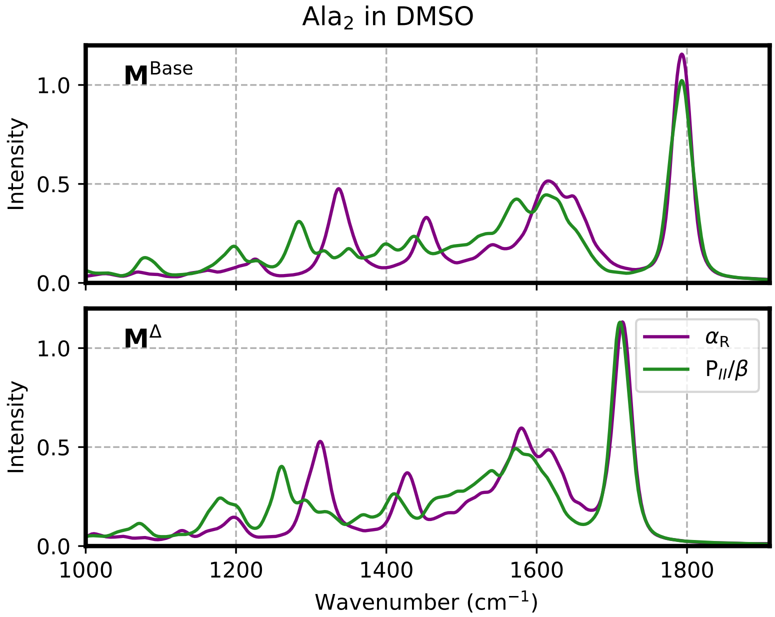

Figure S15: IR spectra in the 1000-1900 cm^{-1} region of Ala$_2$ in DMSO obtained from trajectories that remained in the α_R and P_{II}/β conformations. Reference level of theory for the top and bottom panels are ωB97XD/6-31G(d) and B2PLYP-D3/*cc*-pVTZ, respectively.

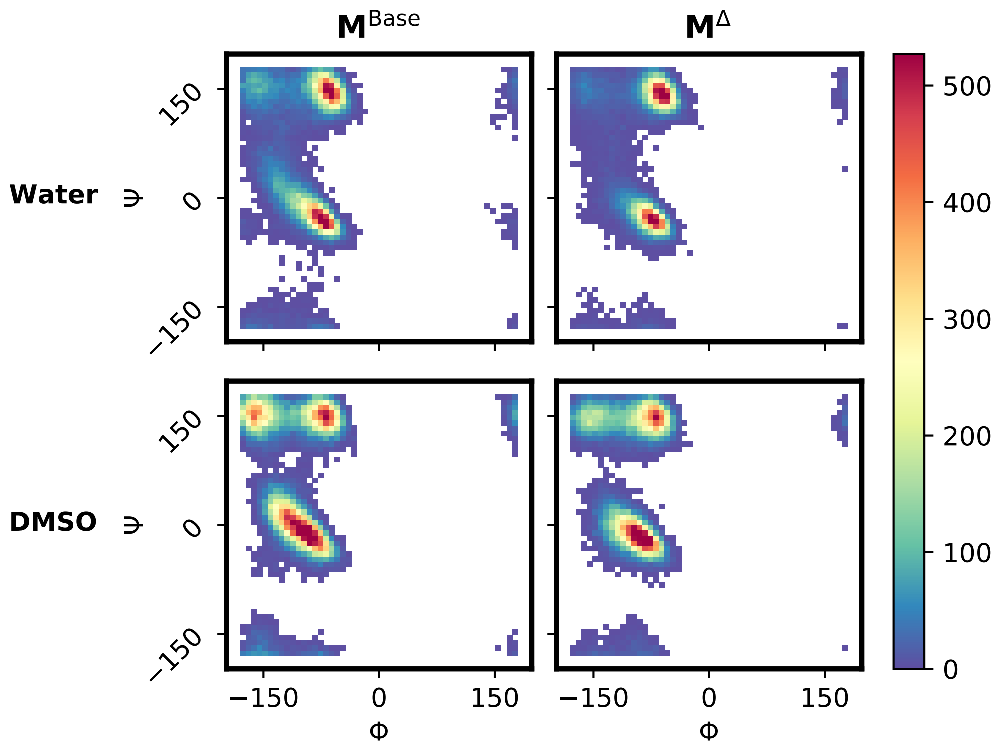

Figure S16: 2D-histogram of the values of the Ramachandran angles during the ML/MM simulations performed in water and DMSO, with $\mathbf{M}^{\mathrm{Base}}$ and $\mathbf{M}^{\Delta}$ models.

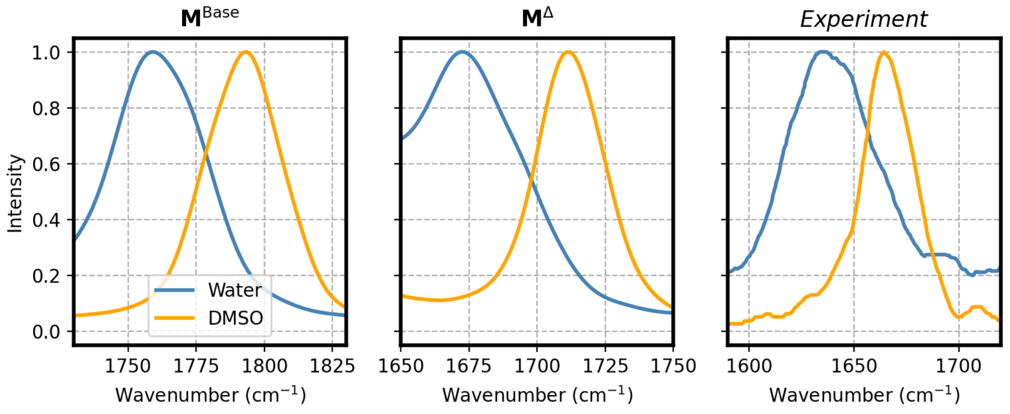

Figure S17: Comparison of IR spectra in the Amide I region for Ala_2 in water and DMSO. Reference levels of theory for the left and middle panels are ωB97XD/6-31G(d) and B2PLYP-D3/*cc*-pVTZ, respectively, whereas in the right panel the experimental spectrum is reported. The experimental spectra are taken from Ref. 6.

S2 Additional tables

Table S1: Timings for the simulations performed on a single V100 GPU with the different models available for each molecule.

		Timings (ns/day)		
Molecule	Environment (n. MM atoms)	$\mathbf{M}^{\text{Base}}$	$\mathbf{M}^{\Delta}$	$\mathbf{M}^{\Delta} + \Delta\mathbf{Env}$
Ura	Vacuum	9.8	8.8	8.4
	Water (3831)	2.0	2.0	2.0
NMA	Vacuum	10.0	8.7	
	Water (3840)	2.0	2.0	
	Chloroform (2790)	2.9	2.8	
Ala_2	DMSO (5370)	1.4	1.4	
	Water (4764)	1.5	1.5	

Table S2: Solvatochromic shift of aqueous uracil IR spectra.

	Solvatochromic shift (cm^{-1})	
	$\Delta\nu_1$	$\Delta\nu_2$
Exp.	-67	30
$\mathbf{M}^{\text{Base}}$	-66	31
$\mathbf{M}^{\Delta}$	-58	25

Table S3: Solvatochromic shift of the IR spectra of NMA in water and chloroform.

	Water (cm^{-1})			Chloroform (cm^{-1})		
	$\Delta\nu_1$ (Amide I)	$\Delta\nu_2$ (Amide II)	$\Delta\nu_3$ (Amide III)	$\Delta\nu_1$	$\Delta\nu_2$	$\Delta\nu_3$
Exp.	-90	68	57	-39	15	-
$\mathbf{M}^{\text{Base}}$	-78	55	45	-18	-5	0
$\mathbf{M}^{\Delta}$	-77	53	41	-17	-6	-1

Table S4: Solvatochromic shift of the Amide I peak for Ala_2 in water and DMSO.

	Solvatochromic shift (cm^{-1})
	Amide I
Exp.	-29
$\mathbf{M}^{\text{Base}}$	-34
$\mathbf{M}^{\Delta}$	-38

References

[1] Szczesniak, M. *et al.* Matrix isolation studies of nucleic acid constituents. 1. infrared spectra of uracil monomers. *J. Am. Chem. Soc.* **105**, 5969–5976 (1983).

[2] Aamouche, A. *et al.* Neutron inelastic scattering, optical spectroscopies and scaled quantum mechanical force fields for analyzing the vibrational dynamics of pyrimidine nucleic acid bases. 1. uracil. *J. Phys. Chem.* **100**, 5224–5234 (1996).

[3] Ataka, S., Takeuchi, H. & Tasumi, M. Infrared studies of the less stable cis form of n-methylformmaide and n-methylacetamide in low-temperature nitrogen matrices and vibrational analyses of the trans and cis forms of these molecules. *J. Mol. Struct.* **113**, 147–160 (1984).

[4] Song, S., Asher, S. A., Krimm, S. & Bandekar, J. Assignment of a new conformation-sensitive uv resonance raman band in peptides and proteins. *J. Am. Chem. Soc.* **110**, 8547–8548 (1988).

[5] DeCamp, M. *et al.* Amide i vibrational dynamics of n-methylacetamide in polar solvents: The role of electrostatic interactions. *J. Phys. Chem. B* **109**, 11016–11026 (2005).

[6] Lee, M.-E., Lee, S. Y., Joo, S.-W. & Cho, K.-H. Amide i bands of terminally blocked alanine in solutions investigated by infrared spectroscopy and density functional theory calculation: Hydrogen-bonding interactions and solvent effects. *J. Phys. Chem. B* **113**, 6894–6897 (2009).
